# Germ cells commit somatic stem cells to differentiation following priming by PI3K/Tor activity in the Drosophila testis

**DOI:** 10.1371/journal.pgen.1009609

**Published:** 2021-12-13

**Authors:** Alice C. Yuen, Kenzo-Hugo Hillion, Ruoxu Wang, Marc Amoyel

**Affiliations:** 1 Department of Cell and Developmental Biology, University College London, London, United Kingdom; 2 Department of Biochemistry and Molecular Pharmacology, New York University School of Medicine, New York, New York, United States of America; New York University, UNITED STATES

## Abstract

How and when potential becomes restricted in differentiating stem cell daughters is poorly understood. While it is thought that signals from the niche are actively required to prevent differentiation, another model proposes that stem cells can reversibly transit between multiple states, some of which are primed, but not committed, to differentiate. In the Drosophila testis, somatic cyst stem cells (CySCs) generate cyst cells, which encapsulate the germline to support its development. We find that CySCs are maintained independently of niche self-renewal signals if activity of the PI3K/Tor pathway is inhibited. Conversely, PI3K/Tor is not sufficient alone to drive differentiation, suggesting that it acts to license cells for differentiation. Indeed, we find that the germline is required for differentiation of CySCs in response to PI3K/Tor elevation, indicating that final commitment to differentiation involves several steps and intercellular communication. We propose that CySC daughter cells are plastic, that their fate depends on the availability of neighbouring germ cells, and that PI3K/Tor acts to induce a primed state for CySC daughters to enable coordinated differentiation with the germline.

## Author summary

Stem cells are unique in their ability to regenerate adult tissues by dividing to provide new stem cells, a process called self-renewal, and cells that will differentiate and maintain tissue function. How and when the daughters that differentiate lose the ability to self-renew is still poorly understood. Self-renewal depends on signals that are provided by the supportive micro-environment, or niche, in which the stem cells reside. It was assumed that simply losing access to this environment and the signals it provides was sufficient to direct differentiation. Here we use the Drosophila testis as a model to show that this is not the case. Instead, differentiation must be actively induced by signalling, and stem cells deprived of all signals can be maintained. Studying the relative timings of the various inputs into differentiation leads us to propose that a series of events ensure appropriate differentiation. First, stem cells receive differentiation-inducing signals that promote a permissive, or primed, state which is reversible and does not preclude self-renewal. The final commitment comes from interacting with other cells in the tissue, ensuring that differentiation always occurs in a coordinated manner among the different cell types composing this tissue.

## Introduction

In tissues with high turnover, stem cells are crucial to maintaining homeostasis by producing new differentiated offspring to replace lost cells. Ensuring long-term tissue maintenance requires that stem cells self-renew, in addition to producing differentiating daughters. Self-renewal capacity was shown early on not to be intrinsic to a stem cell, but to rely instead on the local environment of the cell, termed a niche [1]. The first such niche was identified in the Drosophila ovary [2,3], and our understanding of stem cell-niche interactions has greatly increased since then [4,5]. A common feature of many niches is that the signals they produce actively inhibit differentiation of resident stem cells, exemplified by repression of the differentiation factor *bag of marbles* (*bam*) by niche-derived Bone Morphogenetic Protein (BMP) signals in the Drosophila germline stem cells (GSCs) [6]. This model of niche function implies that the stem cell state is inherently unstable and must be actively maintained. However, in tissues varying from the Drosophila ovary and testis to the mouse intestine [7–10], disrupting certain signalling pathways results in the accumulation of stem cells, implying that stem cell differentiation requires active signalling.

In parallel to this model of stem cell fate in which niche or differentiation signals are instructive, studies conducting lineage tracing of cells expressing markers of a more differentiated fate have shown that marker expression can fluctuate and does not reflect potential, giving rise to a more nuanced picture of what constitutes stem cell identity [11–16]. This work suggests that stem cell potential is distributed across a population of cells within which different states exist where some cells have a higher propensity, or bias, towards self-renewal and some towards differentiation. How stem cells transition between these states and out of the stem cell pool and the contribution of niche signals to these transitions is poorly understood.

**Fig 1:**
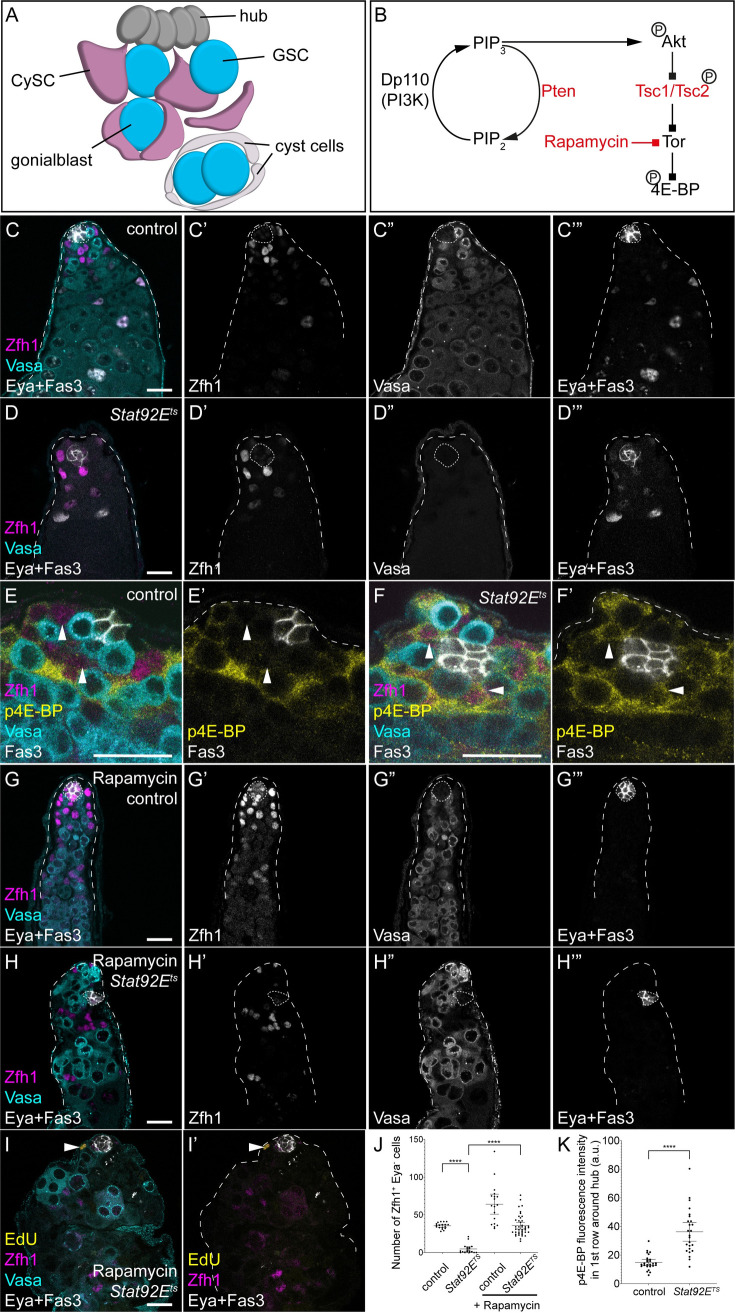
Tor is required for cyst cell differentiation in the absence of the self-renewal signal JAK/STAT. A. Diagram representing the apical tip of the Drosophila testis. Hub cells (dark grey) support two stem cell populations: GSCs (blue) and CySCs (magenta). GSCs divide to give rise to gonialblasts that are wrapped by two CySC daughters. These differentiate into cyst cells (white) and support the encysted germline. B. Simplified schematic of the PI3K/Tor pathway. Activated PI3K phosphorylates Phosphatidylinositol 4,5-bisphosphate (PIP_2_) into Phosphatidylinositol (3,4,5)-trisphosphate (PIP_3_), which results in the phosphorylation and activation of Akt. In turn, Akt phosphorylates Tsc2 to lead to Tor activation. Active Tor phosphorylates 4E-BP, which is a readout for pathway activity. Pten, Tsc1 and Tsc2 in red are negative regulators of the pathway, while the drug rapamycin inhibits Tor activity. C,D. Control and *Stat92E*^*ts*^ animals shifted to 29°C for 7 days showing loss of both CySCs and GSCs in the *Stat92E*^*ts*^ mutant. Zfh1 (magenta, single channel C’,D’) labels CySCs and early daughters, Vasa (cyan, single channel C”,D”) labels the germline, and Eya and Fas3 (white, single channel C”’,D”’) label differentiated cyst cells and the hub, respectively. E,F. p4E-BP is upregulated early in CySCs upon loss of JAK/STAT signalling. In controls (E), high levels of p4E-BP (yellow) are detected in Zfh1-positive (magenta) cells two rows away from the hub (Fas3-positive, white). In *Stat92E*^*ts*^ mutants shifted for 6 h, high p4E-BP is visible in CySCs contacting the hub (arrowheads). Vasa labels the germline in cyan and shows GSCs contacting the hub in both conditions. G-I. Rapamycin feeding inhibits CySC differentiation even in *Stat92E* mutants. In controls (G), rapamycin feeding results in Zfh1-expressing cells (magenta, G’) being found throughout the testis, and no Eya-positive cells (white, G”‘, compare to C”‘). Rapamycin prevents the loss of Zfh1-expressing CySCs in *Stat92E*^*ts*^ mutants after 7 days at 29°C (H), and no Eya-expressing cells are visible. I. EdU incorporation (yellow) is detected in a Zfh1-expressing cell (arrowhead). Vasa (cyan) labels the germline. J. Quantification of the number of Zfh1-positive, Eya-negative CySCs in control and *Stat92E*^*ts*^ mutants, raised on control food or with rapamycin. **** denotes P<0.0001, as determined by Kruskal-Wallis and Dunn’s multiple comparisons test. K. Quantification of p4E-BP fluorescence intensity in CySCs, identified as Zfh1-positive cells one cell diameter from the hub. Fluorescence in CySCs significantly increased in *Stat92E*^*ts*^ testes shifted to the restrictive temperature for 6 h. **** denotes P<0.0001 as determined by Student’s t-test. Lines in J and K indicate mean and 95% confidence interval. The hub is indicated with Fas3 expression or a dotted line. Scale bar in all panels represents 20 μm.

To probe how and when stem cell daughters commit to differentiation, we study the somatic lineage in the Drosophila testis. The testis stem cell niche, called the hub, is composed of approximately 10–12 quiescent cells that support two stem cell populations (Fig 1A) [17]. GSCs adhere tightly to the hub and divide with invariantly oriented divisions, generating in most cases two daughters with asymmetric fates. The differentiating daughter, known as a gonialblast, undergoes 4 rounds of division with incomplete cytokinesis to form a cyst which matures into spermatocytes. The second population, somatic cyst stem cells (CySCs), gives rise to post-mitotic differentiating cells, called cyst cells. These tightly associate with a gonialblast in a 2:1 ratio, ensheathing the developing cyst and eventually sealing off the germline from the environment (Fig 1A) [18–21]. Coordination between the lineages and support from the soma are essential for germ cell differentiation and fertility [18,19,22,23]. Recent work has shown that this coordination is not regulated through synchronised stem cell divisions but instead at the level of co-differentiation of gonialblasts and CySC daughters, when encystment leads to abscission of the gonialblast from the GSC [24]. How the two lineages temporally coordinate their differentiation is difficult to envisage if differentiation is simply a consequence of loss of niche signals. CySCs rely primarily on activity of the Janus Kinase and Signal Transducer and Activator of Transcription (JAK/STAT) pathway for self-renewal [25], while additional signals including the Hedgehog (Hh), Hippo (Hpo), Mitogen-Activated Protein Kinase (MAPK) and Slit/Robo pathways regulate proliferation and niche occupancy [26–31]. BMPs produced by the hub and the CySCs maintain GSC self-renewal, and do so at least in part by directly repressing expression of the differentiation factor *bam*, supporting the model in which niche-derived signals repress differentiation [32,33].

In the cyst lineage, however, clonal assays have revealed that CySC divisions produce equipotent daughters which stochastically self-renew or differentiate and that CySC replacement at the niche by neighbours is a frequent event [28]. CySCs are identified by contact with the niche and progression through the cell cycle, while differentiated cells are post-mitotic; mitoses are only observed at the hub [34,35]. CySCs express the transcription factor Zn finger homeodomain 1 (Zfh1), which is necessary and sufficient for self-renewal [25]. Zfh1 expression is downregulated during differentiation and cyst cells begin expressing the cyst cell marker Eyes Absent (Eya) [36]. Zfh1 expression labels a larger pool of cells than actually contact the hub, suggesting that CySC potential may extend beyond cells with direct hub contact. Consistently, although CySC divisions result in only one cell inheriting the attachment to the hub [35], clonal experiments show that both daughters of a CySC division can gain access to the hub [28]. Indeed, recent work tracing cells lacking hub cell contact showed that they can give rise to labelled CySCs [37]. Finally, cyst cell differentiation requires signalling through the Phosphoinositide 3-kinase (PI3K)/Target of Rapamycin (Tor) pathway (Fig 1B) [10,38,39]. High levels of PI3K and Tor activity are detected in Zfh1-positive cells that do not contact the hub, suggestive of a role during early stages of differentiation. Indeed, CySCs deficient for Tor or PI3K pathway activity maintain Zfh1 expression and fail to express Eya, while clones with gain-of-function mutations in the pathway are not maintained at the hub [10,28,39]. Thus, cyst cell differentiation is actively induced by signalling through PI3K/Tor, yet how the differentiation of cyst cells is temporally regulated to ensure coordination with the germline is not understood.

Here we set out to understand how cyst cell differentiation is regulated by addressing the role of PI3K/Tor signalling in cyst cells. We show that CySC differentiation requires Tor activity even in the absence JAK/STAT signalling, implying that differentiation is not a default state in the absence of niche-derived self-renewal signals. Conversely, the sufficiency of PI3K/Tor to drive differentiation is relative: clonal increases in PI3K/Tor drive differentiation while lineage-wide increases do not. These observations are consistent with a model in which high PI3K/Tor activity primes cells for differentiation but is not sufficient for commitment; clones with artificially increased PI3K/Tor activity are in a persistent primed state and more likely to differentiate. Furthermore, we show that the ability of increased PI3K/Tor to promote differentiation requires the presence of the germline. We propose that CySC differentiation into cyst cells occurs in reversible steps: loss of niche access and self-renewal signals, followed by acquisition of differentiation competence driven by PI3K/Tor activity, before the final commitment to differentiation which occurs upon interaction with a gonialblast. Our work suggests that actively regulating differentiation in this way is an important mechanism for achieving coordination across lineages.

## Results

### Tor is required for cyst cell differentiation in the absence of JAK/STAT signalling

In the somatic cyst lineage of the testis, JAK/STAT signalling is both necessary and sufficient to maintain CySC fate, while PI3K/Tor activity is required for cyst cell differentiation [10,25]. We sought to understand how differentiation is initiated upon withdrawal of JAK/STAT signalling, and whether Tor is required in this process.

We used a temperature-sensitive mutant for the sole Drosophila STAT, *Stat92E*, to deplete JAK/STAT activity in adult males. In testes from these mutants, as described previously [40,41], both GSCs and CySCs were rapidly lost to differentiation. By 7 days at the restrictive temperature (29°C), no GSCs were present at the hub and few, if any, CySCs were observed, defined as cells expressing Zfh1 and negative for expression of the differentiation marker Eya (Fig 1D, compare to control in Fig 1C, quantified in Fig 1J, P<0.0001, Kruskal-Wallis test with Dunn’s multiple comparisons, N = 18 for control, N = 19 for *Stat92E^ts^*). To characterise the events involved in initiating differentiation, we examined testes from *Stat92E^ts^* animals prior to the stem cells being lost to differentiation. Previous work has shown that 16 h after shifting to the restrictive temperature, GSCs are still present [41]. We counted Zfh1-expressing, Eya-negative cells at an earlier time point after shifting to the restrictive temperature, reasoning that CySCs would also be present. Indeed, 6 h after shifting to 29°C, we counted 33.8 ± 0.8 CySCs in *Stat92E/+* heterozygous controls compared to 35.4 ± 1.0 in *Stat92E^ts^* animals (Fig 1E and 1F, P<0.24, Mann-Whitney test, N = 26 and N = 25, respectively). We used an antibody recognising phosphorylated eIF4E-Binding Protein (p4E-BP) to monitor Tor activity in *Stat92E^ts^* testes at this early timepoint after Stat depletion, since this is a well-established readout of Tor activity and was shown to require Tor activity in the testes [10,42,43]. In control *Stat92E/+* heterozygous animals shifted to 29°C for 6 h, p4E-BP was highly expressed in the second row of somatic cells from the hub, and lower in the first row of CySCs that directly contact the hub (Fig 1E, arrowheads). In contrast, in *Stat92E^ts^* mutant animals, the pattern of p4E-BP changed so that high p4E-BP was now detected in somatic cells directly adjacent to the hub (Fig 1F, arrowheads). We quantified the fluorescence intensity in the CySCs immediately contacting the hub and observed a statistically significant increase in *Stat92E^ts^* mutants compared to control (Fig 1K, P<0.0001, t-test). Other pathways have been shown to be involved in CySC fate decisions, in particular Hedgehog (Hh) signalling, which determines the size of the stem cell pool but is neither required nor sufficient to prevent differentiation [27–29]. We asked whether Hh signalling also influenced Tor signalling by analysing p4E-BP expression in testes over-expressing the repressive (Ci^R^) or activated form (Ci^Act^) of the Hh effector Cubitus interruptus (Ci). Although these manipulations affected the number of Zfh1-positive CySCs, they did not result in changes in the pattern of p4E-BP, which remained excluded from CySCs in contact with the hub (S1A–S1C Fig, white arrowheads) and enriched in daughters leaving the niche (S1A–S1C Fig, yellow arrowheads). These results show that Tor activity is induced in CySCs that will differentiate upon depletion of JAK/STAT, and precedes detectable changes in morphology and marker expression.

Since previous work showed that Tor activity is required for differentiation [10,39] and since Tor activity is upregulated upon JAK/STAT loss, we asked whether Tor is required for the CySC differentiation that occurs following loss of Stat92E activity. In other words, we tested whether cyst cell differentiation is a default state in cells lacking JAK/STAT activity, or whether differentiation is induced by Tor. As shown above, loss of JAK/STAT by shifting *Stat92E^ts^* animals to the restrictive temperature for 7 days results in almost complete loss of CySCs and GSCs (Fig 1C and 1D). Conversely, inhibiting Tor activity by feeding the inhibitor Rapamycin led to Zfh1-positive cells being found away from the hub which did not express Eya (Fig 1G), indicating a block in differentiation. When *Stat92E^ts^* mutant animals were fed Rapamycin and shifted to the restrictive temperature, testes contained Zfh1-positive cells distant from the hub and few Eya-positive cells (Fig 1H), indicating that these testes accumulated undifferentiated CySCs. In addition, Vasa-positive GSCs were present in these testes, although since the role of Tor signalling in germ cells has not been established, it is unclear whether this is an indirect consequence of restoring CySC function or an autonomous effect on GSCs. To confirm that the ectopic Zfh1-positive cells are indeed CySCs, we assessed whether any cells incorporated the nucleotide analogue EdU, as CySCs are the only proliferating somatic cells in the testis. We observed EdU incorporation in Zfh1-positive cells in *Stat92E^ts^* animals fed with Rapamycin (Fig 1I), indicating that Rapamycin feeding maintained CySCs even in the absence of JAK/STAT activity.

Altogether, these results demonstrate that cyst cell differentiation requires active signalling through Tor, even in the absence of the main self-renewal factor, JAK/STAT. This finding suggests that CySC identity can be maintained in the absence of niche signals, as long as differentiation signals are also inhibited.

### Clonal increases in Tor activity promote cyst cell differentiation

These results together with previously published work [10,38,39] show that Tor activity is absolutely necessary for cyst cell differentiation. We next asked whether increases in Tor activity directly induce differentiation or whether it is merely permissive for differentiation. To this end, we used mitotic recombination to generate single cell CySC clones with gain-of-function mutations in the PI3K/Tor pathway. Since CySCs are the only mitotic somatic cells, all labelled somatic cells are the result of a CySC division. We examined clones at 2 days post clone induction (dpci) to verify induction rates, and at 7 dpci to assess the persistence of these clones, which directly reflects the self-renewal and differentiation capacity of the labelled cells.

**Fig 2:**
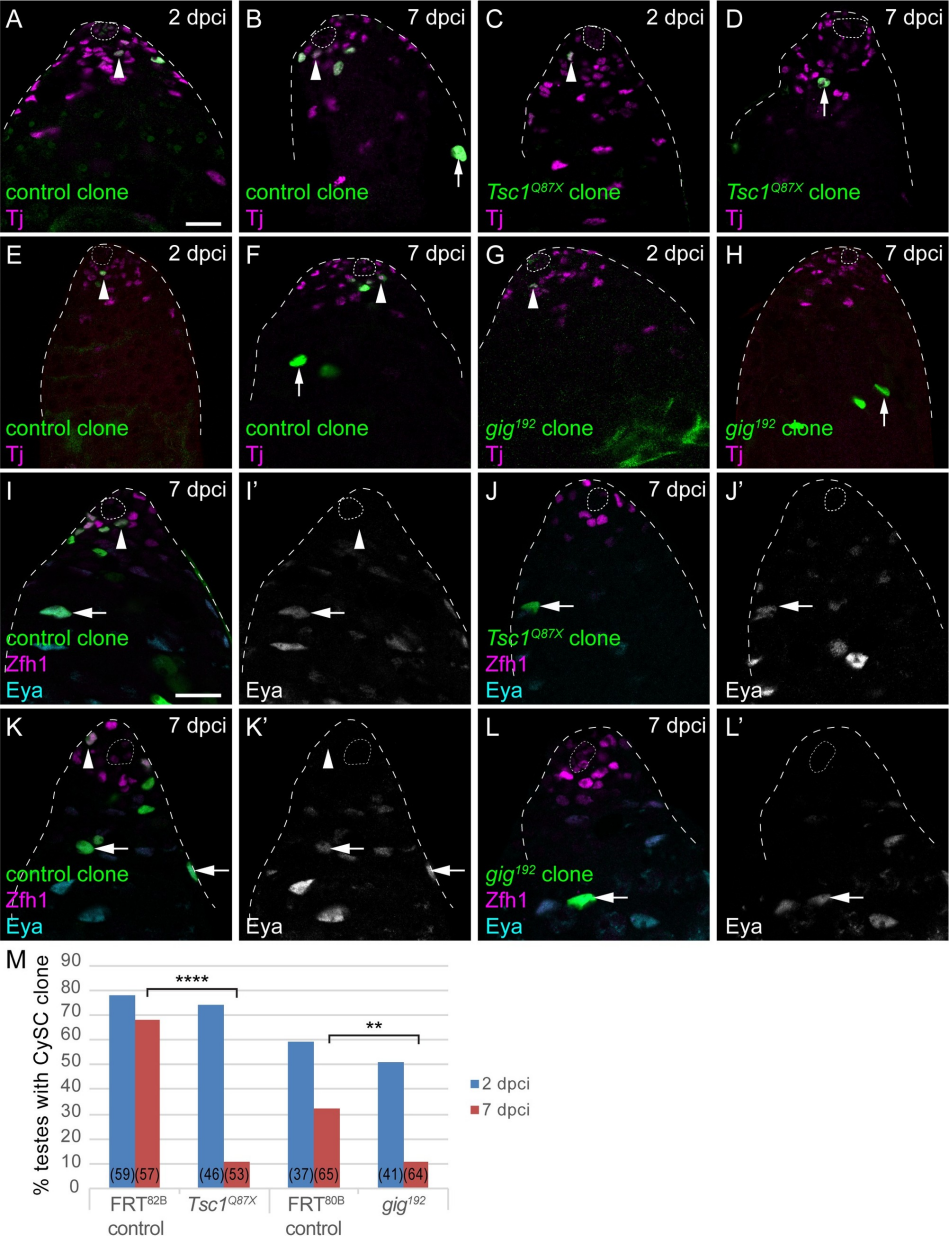
Clonal gain-of-function in Tor activity is sufficient to induce CySCs to differentiate. A-H. Positively-labelled CySC clones generated by MARCM. Control clones generated with *FRT*^*82B*^ (A,B) or *FRT*^*80B*^ (E,F) were observed at 2 days post clone induction (dpci) and at 7 dpci to assess clone induction and maintenance rates, respectively. CySCs (arrowheads) were identified as cells expressing Tj (magenta) and in the first row from the hub, differentiated cyst cells (arrows) have larger nuclei and are found away from the hub. *Tsc1* mutant (C,D) or *gig* (Tsc2, G,H) CySC clones could be induced and were present at 2 dpci (C,G) but were rarely observed by 7 dpci (D,H), where differentiated cells were labelled. I-L. Clones (green) in testes labelled with Zfh1 (magenta) and Eya (cyan, single channels in I’-L’). In controls at 7 dpci (I,K), clones contained both Zfh1-expressing CySCs (arrowheads) and Eya-positive cyst cells (arrows). However, *Tsc1* mutant (J) or *gig* mutant (L) clones had differentiated and were composed of Eya-expressing differentiated cells. The hub is outlined with a dotted line. Scale bar in panel A panels represents 20 μm for A-H and scale bar in panel I panels represents 20 μm for I-L. M. Graph showing the percentage of testes containing a CySC clone at 2 (blue) and 7 (red) dpci. The number of testes examined is showed in parentheses for each column. Statistical significance was determined using Fisher’s exact test, **** denotes P<0.0001, ** denotes P<0.005.

In controls, clones were reliably induced at 2 dpci (Fig 2A and 2E), and many of these clones were present a week after induction (Fig 2B and 2F). Clones at 7 dpci consisted of both CySCs, identified as the first row of Traffic Jam (Tj)-positive somatic cells from the hub (Fig 2B and 2F arrowheads), and differentiated cyst cells, which were displaced from the hub and grew larger (Fig 2B and 2F arrows). Clones mutant for genes encoding components of the Tor repressive Tuberous Sclerosis Complex (TSC), *Tsc1* or *gigas* (*gig*, the Drosophila homologue of *Tsc2*) were also recovered at 2 dpci (Fig 2C and 2G, arrowheads), indicating that mutant CySCs could be induced. However, by 7 dpci, labelled CySC clones were rarely recovered (Fig 2D, 2H and 2M, P<0.0001 for *Tsc1* compared to control, P<0.005 for *gig* compared to control), implying that the mutant cells had differentiated. Indeed, some testes with no CySC clones contained labelled cells distant from the hub, consistent with a mutant CySC that had differentiated and left the niche (Fig 2D and 2H, arrows). We obtained similar results when generating clones mutant for the PI3K inhibitor, *Pten* (see below), confirming previous observations that *Pten*, and *Tsc1* mutant and wild type clones over-expressing the catalytic subunit of PI3K, Dp110, were not maintained at the niche and differentiated [28].

To confirm that *Tsc1* and *gig* mutant clones were lost to differentiation, we stained these clones with antibodies against Zfh1 and Eya, to mark CySCs and differentiated cyst cells. While control clones were composed of both Zfh1-expressing CySCs and Eya-positive cyst cells (Fig 2I and 2K, arrowheads and arrows, respectively), *Tsc1* and *gig* homozygous mutant clones consisted mostly of Eya-positive cells (Fig 2J and 2L, arrows). We asked whether these clones might be lost from the niche due to decreased adhesion. We stained clones with an antibody against Armadillo (Arm), the Drosophila homologue of β-Catenin. In both control and *Tsc1* mutant clones, Arm was detected along the outline of the clone, in the extension contacting the hub (S2A and S2B Fig arrows). Moreover, the same analysis showed that *Tsc1* mutant clones engulfed and associated closely with germ cells (S2B Fig, red arrowhead), indicating that the mutant cells could interact with gonialblasts and differentiate. Finally, we tested whether *Tsc1* and *gig* mutant clones were lost due to increased cell death by staining with an antibody against cleaved Death Caspase-1 (Dcp-1), which labels apoptotic cells. We observed no increase in Dcp-1 staining in mutant clones compared to control (S2D and S2E Fig, 3/13 control clones containing a Dcp-1-positive cell compared to 2/9 *gig* mutant clones, and 1/20 control clones compared to 1/9 *Tsc1* mutants, P<0.45, Fisher’s exact test).

In sum, CySC clones with hyper-activated PI3K or Tor signalling self-renew less well than control CySC clones, and are lost from the niche through differentiation. This implies that PI3K/Tor activity is either deleterious for self-renewal, or is sufficient to promote differentiation. Since the results described in prior work [10,38,39] and in Fig 1 argue that Tor is necessary for differentiation, the most parsimonious interpretation is that clonally increasing PI3K/Tor activity induces CySCs to differentiate into cyst cells.

### Lineage-wide increases in Tor activity do not affect CySCs

The data presented so far appeared to support a model in which, as daughters of a CySC division leave the hub and are out of the range of JAK/STAT signalling, they upregulate PI3K/Tor activity to differentiate. This is consistent with the observation that high levels of the Tor target p4E-BP are detected in the second row of Zfh1-expressing cells (Fig 1E and [10,39]). However, although CySCs divide such that only one daughter cell inherits the hub attachment [35], clonal analysis showed that both daughter cells are equipotent [28], suggesting that cells away from the hub maintain the ability to self-renew. Moreover, lineage tracing using *spichthyin* (*spict)-Gal4*, which is expressed in Zfh1-positive cells in the second row, indicate that these cells do give rise to CySCs [37], raising the possibility that cells with high PI3K/Tor do not necessarily differentiate. We confirmed that indeed, *spict-Gal4*-driven GFP and high levels of p4E-BP do indeed colocalise in Zfh1-expressing cells (S3 Fig, arrows). Thus, cells with high PI3K/Tor are not necessarily committed to differentiate.

**Fig 3:**
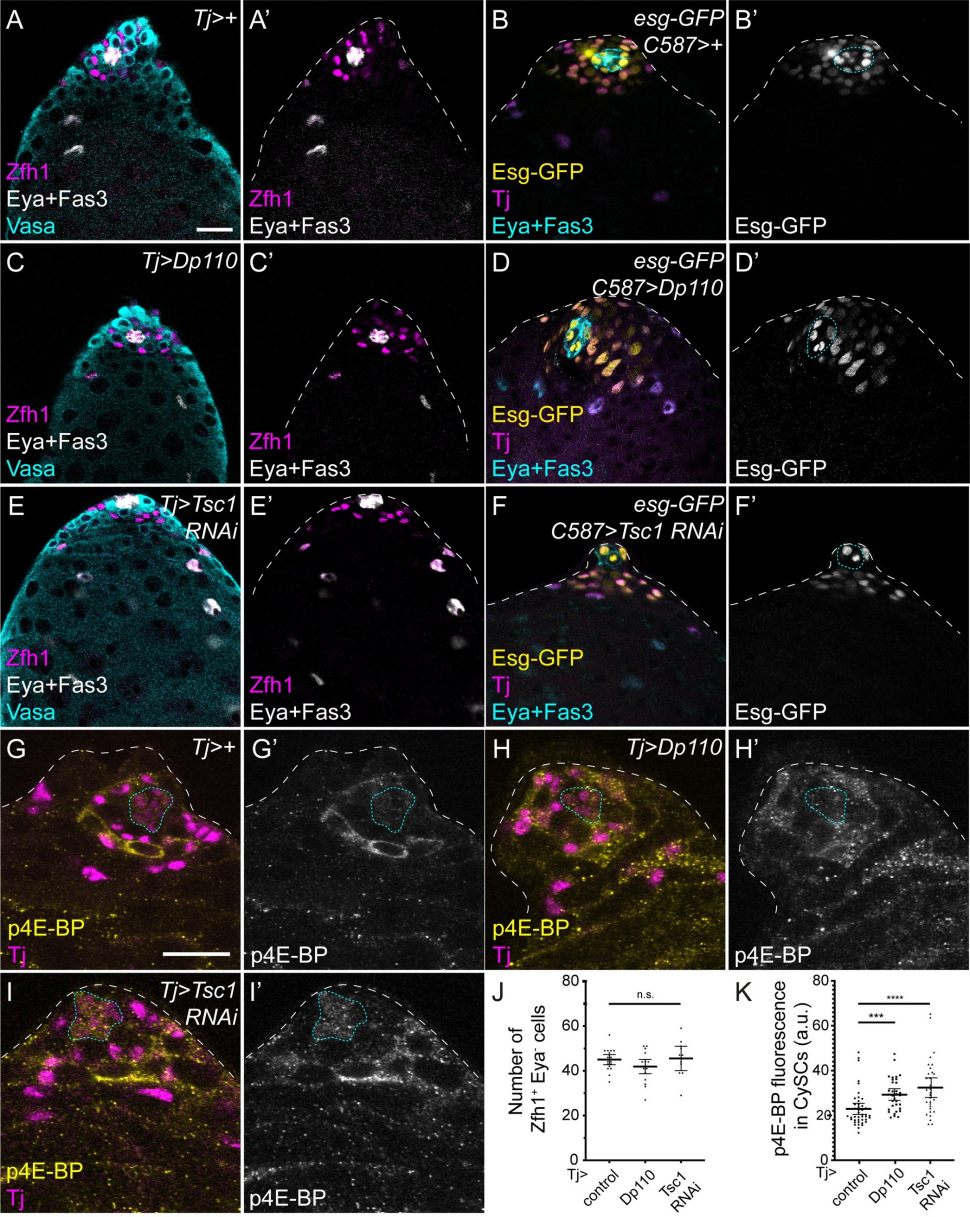
Lineage-wide PI3K/Tor hyperactivation does not induce CySC loss. Control (A,B,G), Dp110 over-expression (C,D,H), or Tsc1 knockdown (E,F,I) in the somatic lineage using *tj-Gal4* (A,C,E,G-H) or *C587-Gal4* (B,D,F). A,C,E. Zfh1 (magenta) labels CySCs and early daughters, Eya and Fas3 (white) label differentiated cyst cells and the hub, respectively and germ cells are labelled with Vasa (cyan). B,D,E. Esg-GFP expression is shown in yellow, Tj (magenta) labels CySCs and differentiating cyst cells and Eya and Fas3 are shown in cyan. Zfh1- or Esg-expressing CySCs are observed upon PI3K or Tor hyperactivation in all conditions. G-I. p4E-BP staining (yellow) is increased upon Dp110 over-expression (H) or Tsc1 knockdown (I). Tj labels the somatic lineage (magenta). J. Graph showing the number of Zfh1-positive, Eya-negative CySCs upon Tor pathway hyperactivation. No significant changes were observed (Kruskal-Wallis, P<0.36, N = 19 for *Tj>+*, N = 18 for *Tj>Dp110* and N = 11 for *Tj>Tsc1 RNAi*). K. Quantification of p4E-BP fluorescence intensity in CySCs surrounding the hub. Over-expression of Dp110 or knockdown of Tsc1 significantly increases p4E-BP intensity (Kruskal-Wallis and Dunn’s multiple comparisons, P<0.0003 for *Tj>Dp110* vs *Tj>+*, P<0.0001 for *Tj>Tsc1 RNAi* vs *Tj>+*, N = 43 for *Tj>+*, N = 33 for *Tj>Dp110* and N = 32 for *Tj>Tsc1 RNAi*). Lines in J and K indicate mean and 95% confidence interval. The hub is outlined with a blue dotted line. Scale bar in panel A panels represents 20 μm for A-F and scale bar in panel G panels represents 20 μm for G-I.

As a further test for the sufficiency of increased PI3K/Tor in promoting CySC differentiation, we hyper-activated signalling in all CySCs using the *traffic jam* (*tj*)*-Gal4* driver. We predicted that, if increasing signalling drives differentiation, many or most CySCs would be lost to differentiation, resulting in testes containing few or no Zfh1-expressing cells, similar to loss-of-function of JAK/STAT signalling. Surprisingly, over-expression of Dp110 or knockdown of *Tsc1* using *tj-Gal4* resulted in testes that were indistinguishable from control (compare Fig 3A, 3C and 3E). There was no significant change in the number of Zfh1-positive and Eya-negative CySCs (Fig 3J, 45±1.1 in control compared to 41.9±1.5 and 45.5±2.4 in Dp110-expressing or *Tsc1* knockdown testes, respectively, N = 19,18,11, P<0.25). To rule out experimental artefacts, we tested whether these constructs could induce ectopic PI3K and Tor activity. First, we used *engrailed (en)-Gal4* to drive expression of *UAS-Dp110* or *UAS-Tsc1 RNAi* in the posterior compartment of larval wing imaginal discs, where PI3K and Tor are well-established regulators of growth during development. Compared to the anterior compartment of the same discs, Dp110 over-expression and *Tsc1* knockdown both led to reduced density of cell nuclei, reflecting increased growth (S4A–S4D Fig). We also used established readouts for the activity of both pathways: phosphorylated Akt1 (pAkt) for PI3K signalling, and phosphorylated S6 Kinase (pS6k) for Tor signalling. Over-expressing Dp110 in wing discs led to a robust increase in pAkt (S4B Fig) and knockdown of *Tsc1* resulted in increased pS6k staining in the posterior compartment (S4D Fig). Finally, we tested for increased pathway activity in testes under these conditions. *Tsc1* knock down or Dp110 over-expression in the cyst lineage resulted in increased p4E-BP staining (Fig 3H and 3I, compare with Fig 3G, quantified in Fig 3K), indicating that these manipulations elevate Tor activity, even if they do not affect the number of CySCs.

To confirm that CySCs were indeed still present under conditions of PI3K or Tor hyperactivation, we used an enhancer trap in the *escargot* locus, *esg-GFP* (Fig 3B, 3D and 3F) to label CySCs and early cyst cells [44]. We observed no differences in Esg-GFP expression when Tor was hyperactivated compared to controls (Fig 3B, 3D and 3F). We also assayed for proliferation by EdU incorporation, since CySCs are the only proliferating somatic cells in the testes. Zfh1-positive cells that incorporated EdU were readily observed when PI3K or Tor were hyperactivated (S5A, S5C and S5E Fig). Finally, we assessed JAK/STAT signalling under these conditions, and observed no changes in the pattern of Stat92E labelling (S5B, S5D and S5F Fig), or in the expression of Chinmo, which is downregulated during cyst cell differentiation [45].

We therefore conclude that increasing PI3K or Tor activity in CySCs is sufficient to drive differentiation when such manipulations are carried out in clones, but not in the entire CySC population.

### Differentiation of CySCs with increased PI3K activity depends on signalling in neighbouring cells

The experiments described above suggest a model in which PI3K/Tor activity induces a permissive state for differentiation, but does not commit cells to differentiation. We propose that PI3K/Tor activity acts as a licensing factor to permit differentiation, and therefore increases the likelihood of differentiation, but upon exposure to hub-derived signals, cells experiencing high PI3K/Tor can self-renew. This model predicts that in clonal experiments, increasing PI3K/Tor results in an increased probability of differentiation and eventual loss of the clone from the stem cell pool, while in lineage-wide gain-of-function, all cells are licensed to differentiate but only a proportion will commit to a differentiated fate.

**Fig 4:**
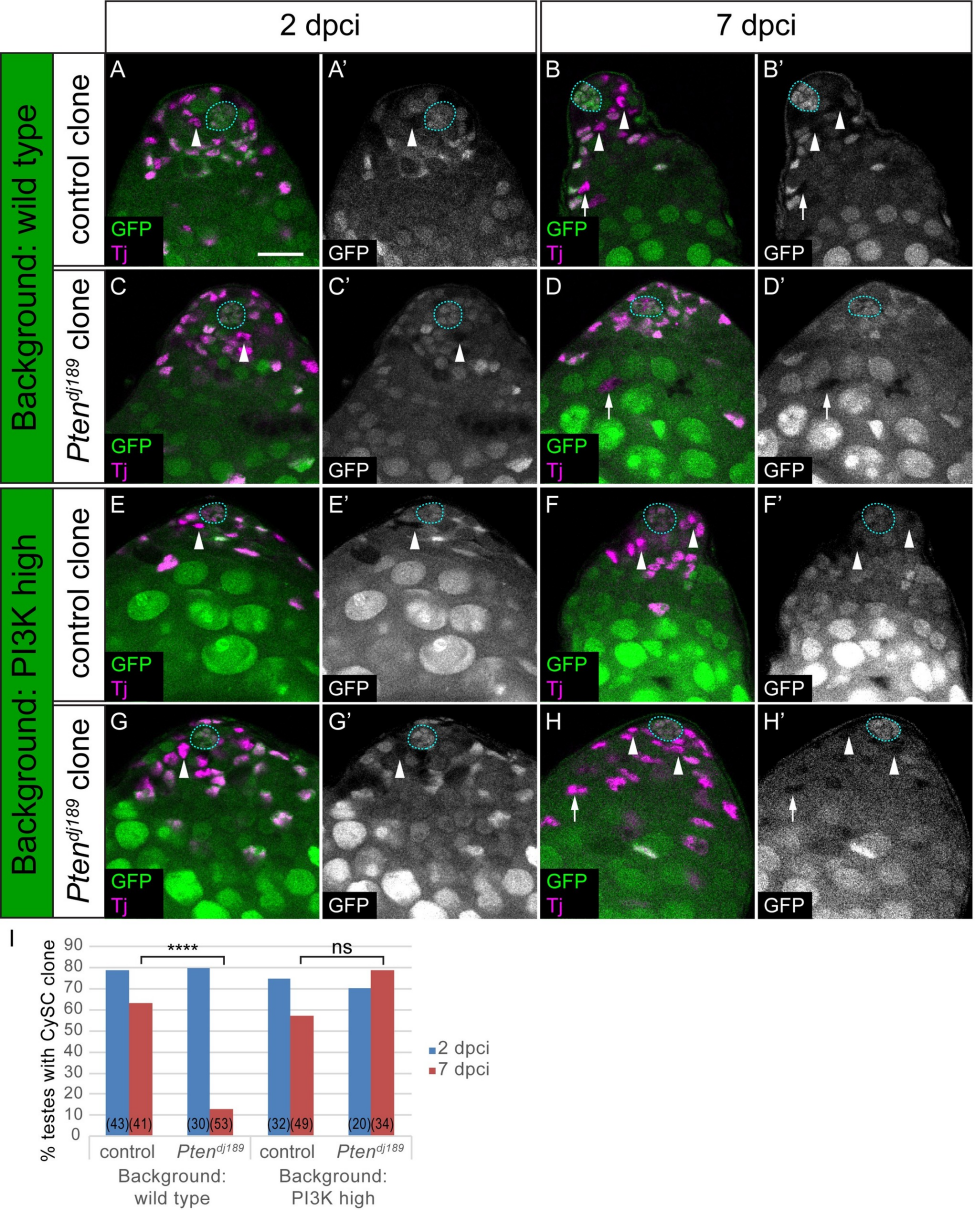
Differentiation of PI3K-high CySCs depends on signalling levels in neighbouring cells. Control (A,B,E,F) and *Pten* mutant (C,D,G,H) clones induced by mitotic recombination and marked by loss of GFP expression, generated either in a control background (*C587-Gal4>+*, A-D) or in a background with elevated PI3K activity (*C587-Gal4>Dp110*, E-H). CySCs (indicated with arrowheads) were identified as Tj-positive (magenta) nuclei one cell diameter from the hub (outlined with a blue dotted line). While *Pten* mutant CySC clones were rarely recovered at 7 dpci, mutant cyst cells were observed (D, arrow) indicating differentiation of the clones. By contrast, *Pten* mutants self-renewed and CySC clones were recovered when PI3K activity was elevated (H, arrowheads). Scale bar in all panels represents 20 μm. I. Graph showing the percentage of testes containing a CySC clone at 2 (blue) and 7 (red) dpci. The number of testes examined is showed in parentheses for each column. Statistical significance was determined using Fisher’s exact test, **** denotes P<0.0001, ns: not significant.

We took two approaches to test the model that PI3K/Tor signalling primes cells for differentiation. First, one prediction is that the differentiation of *Pten* mutant clones is due to higher PI3K/Tor signal in the mutant clones causing them to remain in a permissive state for differentiation, and not an intrinsic inability to self-renew. We reasoned that the maintenance of *Pten* mutant CySC clones could be rescued by increasing PI3K levels in all CySCs, thereby equalising the differentiation likelihood between *Pten* mutants and neighbours. We generated negatively-marked clones, either wild type or mutant for *Pten*, and assessed whether these clones were observed at 2 dpci and 7 dpci. As previously, control Tj-positive clones lacking GFP were observed in the first row from the hub at both 2 and 7 dpci, indicating that CySC clones were induced and maintained over the course of the experiment (Fig 4A and 4B). In contrast, and consistent with published data [28], *Pten* mutant clones, although induced robustly (Fig 4C), were rarely recovered by 7 dpci (Fig 4D and 4I, P<0.0001). When we used the C587-Gal4 driver to over-express Dp110 in CySCs, wild type clones were recovered at similar rates to control (Fig 4E, 4F and 4I, P<0.7). However, *Pten* mutant clones induced in testes where Dp110 was expressed with *tj*-*Gal4* were recovered as frequently as wild type clones at 7 dpci (Fig 4G–4I, P<0.06) and were significantly rescued compared to *Pten* mutant clones in a control background (P<0.0001). These data indicate that *Pten* mutant CySCs maintain the ability to self-renew but that they are normally prevented from doing so by an increased tendency to differentiate compared to neighbouring cells.

**Fig 5:**
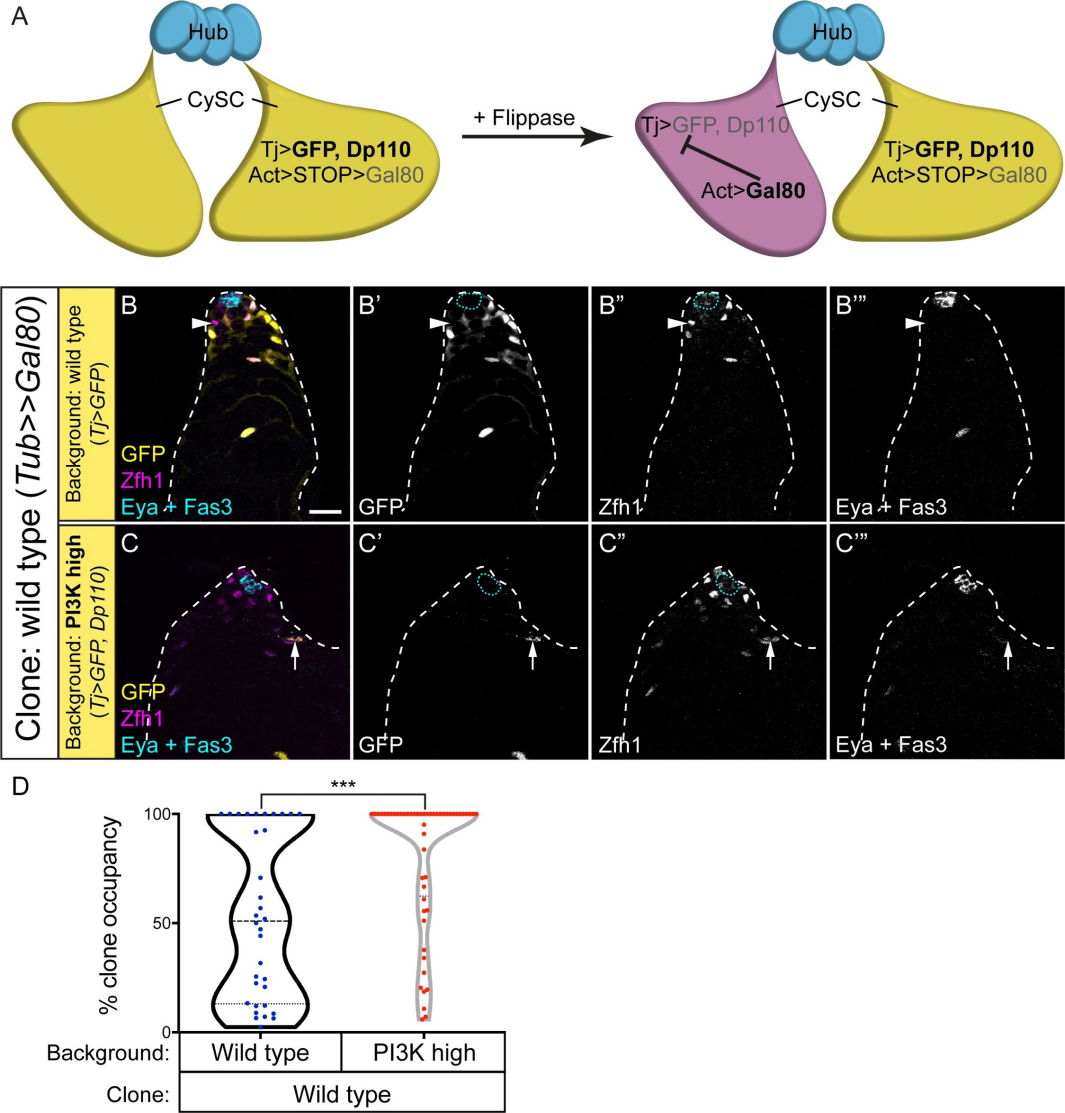
Differences in neighbour PI3K activity levels influences the niche occupancy of wild type clones. A. Schematic explaining the genotypes of clones and background. All CySCs express GFP (yellow) and Dp110 under *tj-Gal4* control while a flipout cassette with an Actin promoter driving Gal80 expression is inactive due to a STOP sequence. Flippase induction excises the STOP cassette, resulting in Gal80 expression which inhibits the activity of Gal4. The resulting cell (magenta) is GFP-negative and does not over-express Dp110. B,C. Wild type Gal80-expressing clones were generated in the cyst lineage that lacked GFP (yellow, single channels in B’,C’). CySCs were labelled with Zfh1 (magenta, single channels in B”,C”), and the hub and cyst cells with Fas3 and Eya respectively (white, single channels in B”’,C”’). B. At 14 dpci, wild type CySC clones (arrowhead) were observed surrounded by GFP-expressing CySCs. C. When GFP-expressing cells also had high PI3K activity, wild type CySC clones occupied a greater proportion of the stem cell pool, and often replaced all GFP-expressing CySCs. Arrow in C points to a GFP-expressing, Eya-positive differentiating cyst cell. Scale bar represents 20 μm. D. Quantification of the clone occupancy for wild type clones in different backgrounds. Clone occupancy is the number of GFP-negative cells as a proportion of all Zfh1-positive cells. Statistical significance was determined using a Mann-Whitney test, *** denotes P<0.0005.

As a second test of the hypothesis that PI3K/Tor activity acts to licence cells for differentiation, we generated mosaic testes containing cells with defined levels of PI3K activity. To ensure that both cell types can both self-renew and differentiate, we generated clones that were wild type in a background where all cells had elevated PI3K activity (Fig 5A, and see Materials and Methods and S1 Table for how clones were generated and relevant genotypes). In this situation, as shown in Fig 3, Dp110 over-expressing CySCs had no detectable phenotype and self-renewed well. However, we predicted that if PI3K levels determined the ability of cells to differentiate, wild type clones would be less likely to differentiate than Dp110-expressing cells, and that over time, wild type cells should come to dominate the CySC pool. We expressed Dp110 together with GFP under the control of *tj-Gal4* and induced clones in this background that expressed the Gal4 inhibitor, Gal80. These clones had no Gal4 activity and were therefore wild type for PI3K, and were labelled by loss of GFP expression. When we generated Gal80-expressing wild type clones in testes expressing only GFP under *tj*-*Gal4* control, we observed a mix of GFP-positive and negative cells with a broad distribution of clone sizes (Fig 5B and 5D), consistent with previous observations that CySCs compete stochastically with each other for niche occupancy, with each stem cell having an equal chance of outcompeting its neighbour or of being displaced from the niche [28]. When we induced Gal80-expressing clones in testes expressing both GFP and Dp110 under *tj*-*Gal4* control, many more GFP-negative cells were observed at 14 dpci (Fig 5C and 5D, P<0.0006). This was not due to increased clone induction rates, as similar numbers of GFP-negative cells were present at 2 dpci (14.7 ± 1.6 GFP-negative cells in control compared to 13.2 ± 2.8 in a Dp110-expressing background, N = 29 and 13, respectively, P<0.58). Thus, the ability of Gal80 clones with wild type levels of PI3K to self-renew was significantly affected by PI3K levels in neighbouring cells: in control conditions, these clones have no obvious advantage; however, when surrounded by PI3K-high cells, they are more likely to self-renew and colonise the niche.

### Germ cells promote cyst lineage differentiation

The experiments described above indicate that PI3K/Tor signalling is required to allow CySCs to differentiate, but that it is not sufficient to commit cells to differentiation. This implies that differentiation itself requires an additional input, and that only a subset of the cells that are primed receive this and go on to differentiate. In the testis, cyst cells co-differentiate with GSC daughters, gonialblasts, such that two cyst cells encapsulate one goniablast. Gonialblasts, therefore, are a good candidate to be the limiting resource for cyst cell differentiation.

**Fig 6:**
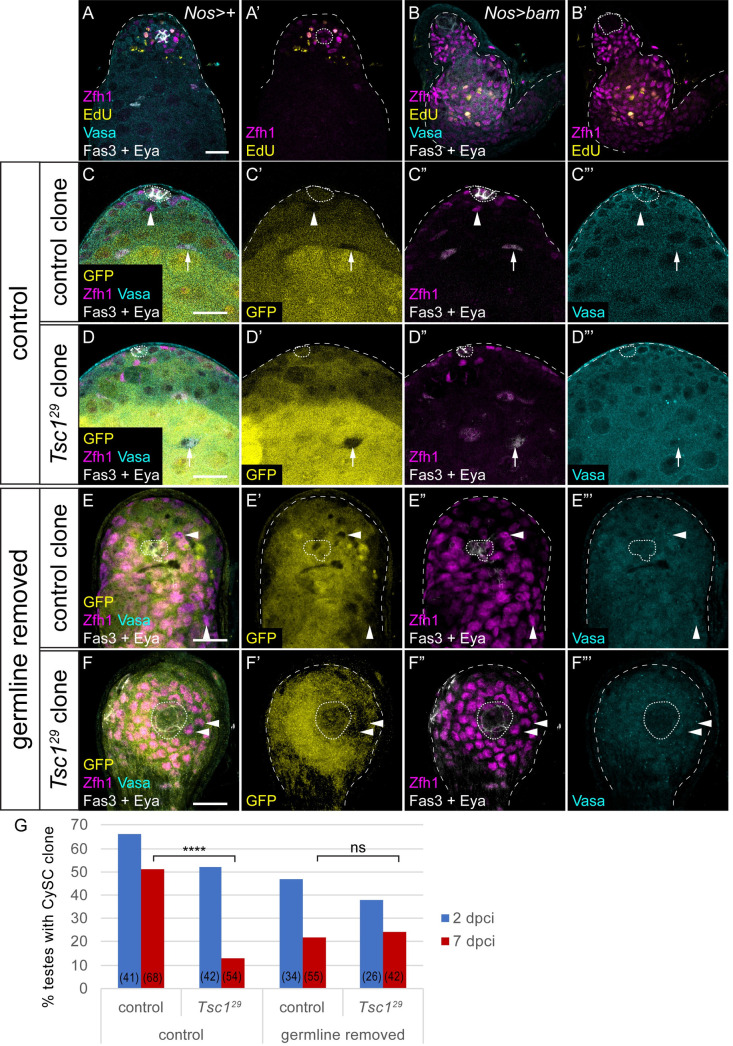
The germline is required for differential PI3K activity to induce differentiation. A,B. Control (A) and germline-ablated (B) testes, showing Zfh1 (magenta) to label CySCs and early daughters, Fas3 and Eya (white) to label the hub and differentiated cyst cells respectively, Vasa (cyan) to label the germline and EdU (yellow) labelling cells replicating DNA. Germline ablation results in loss of Vasa staining, expansion of Zfh1 away from the hub and EdU incorporation in somatic cells away from the hub. C-F. Control (C,E) or *Tsc1* mutant (D,F) negatively-marked clones at 7 dpci. Clones are identified by the absence of GFP (yellow, single channel C’-F’). In testes where the germline (Vasa-positive, cyan, single channel C’”-F”’) is present control CySC clones (C) are recovered and contain both Zfh1-expressing CySCs (arrowheads, magenta) and Eya-positive cyst cells (arrows, white), while *Tsc1* mutant CySCs (D) are rarely observed. By contrast, when the germline is absent (E,F), control and *Tsc1* mutant clones are recovered at similar rates, and both consist of only Zfh1-expressing cells. Scale bar in all panels represents 20 μm. G. Graph showing the percentage of testes containing a CySC clone (Zfh1-positive cells adjacent to the hub) at 2 (blue) and 7 (red) dpci. The number of testes examined is showed in parentheses for each column. Statistical significance was determined using Fisher’s exact test, **** denotes P<0.0001, ns: not significant.

First, we tested whether the presence of a germline affected cyst lineage differentiation. Previous work has shown that agametic testes display ectopically proliferating somatic cells, consistent with excess undifferentiated CySCs being present [46]. We generated testes in which the germline was forced to undergo differentiation, by expressing the germ cell differentiation factor Bam under control of the GSC and early germ cell driver *nanos* (*nos)-Gal4* [47]. Bam expression in the germline resulted in variably penetrant germ cell loss from the niche. In testes completely lacking a germline, we observed ectopic Zfh1-expressing cells many cell diameters away from the hub, and few Eya-positive cells (Fig 6A and 6B). Moreover, we detected many Zfh1-positive cells that were proliferating, as assessed by EdU incorporation (Fig 6B). Additionally, Chinmo expression was maintained away from the hub (S6A and S6C Fig arrows), consistent with a lack of differentiation in these conditions. We tested whether this could be due to ectopic JAK/STAT signalling and found that stabilised Stat92E could be detected up to three cell diameters away from the hub when the germline was ablated (S6B and S6D Fig), suggesting that the absence of GSCs allows further diffusion of the JAK/STAT ligand Unpaired, but is not sufficient to explain ectopic self-renewal distant from the hub (Fig 6B). These data show that loss of the germline results in ectopic CySC-like cells, consistent with published work implicating germ cells in promoting cyst cell differentiation [46].

To test the hypothesis that CySC daughter cells are competing for gonialblasts to co-differentiate with, we induced clones with a gain-of-function mutation in the PI3K/Tor pathway in agametic testes. If the role of PI3K/Tor is indeed to prime CySC daughter cells to encapsulate a gonialblast, the lack of gonialblasts would result in clones with elevated PI3K/Tor remaining undifferentiated. Conversely, if elevating PI3K/Tor was sufficient to drive differentiation independently of the germline, these clones should differentiate, similar to PI3K/Tor gain-of-function clones in wild type testes (Fig 2). Clones mutant for the Tor pathway inhibitor *Tsc1* were readily induced and observed at 2 dpci (Fig 6D and 6G), but differentiated rapidly and few CySC-containing clones were recovered at 7 dpci, compared to control clones (Fig 6C and 6G, P<0.0001). However, when we generated *Tsc1* mutant clones in testes where the germline underwent forced differentiation, CySC clones were now recovered at similar rates to control clones (Fig 6E–6G, P<0.8). Thus, in the absence of germ cells to encyst, CySCs with elevated Tor activity can self-renew similarly to control CySCs. These data imply that Tor does not autonomously promote differentiation, but, together with our previous experiments, show that Tor activity regulates the ability of individual CySC daughter cells to co-differentiate with the germline.

## Discussion

**Fig 7:**
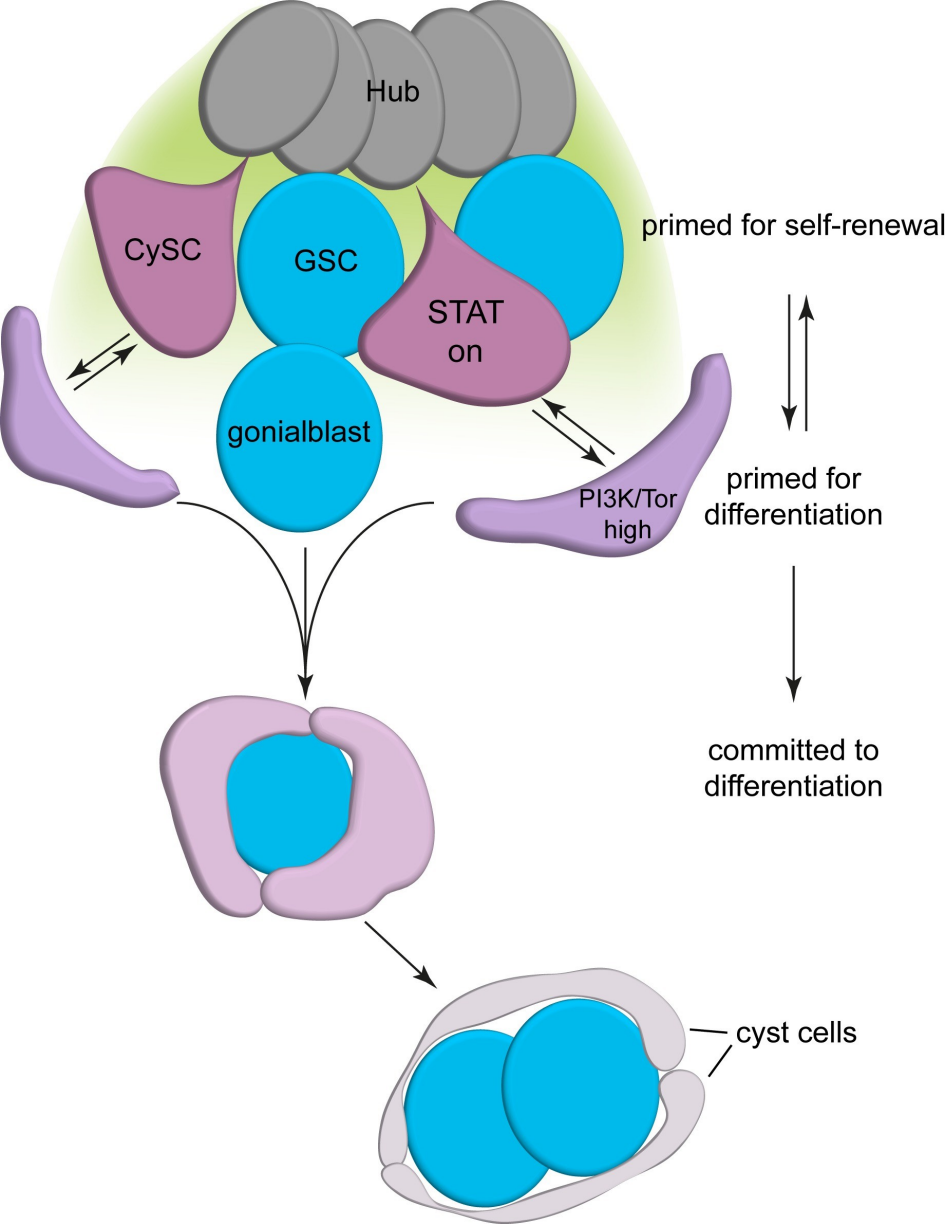
Model of CySC differentiation. CySCs (purple) at the hub (dark grey) receive self-renewal signals (indicated by a green gradient) and are primed for self-renewal. Upon losing access to the hub and experiencing increased PI3K/Tor activity, CySC daughter cells become primed for differentiation, and can interact with goniablasts that arise from GSC divisions. Differentiation priming is reversible, and cells with high PI3K/Tor maintain stem cell potential. Upon interaction with a gonialbast, a cyst forms and the cells co-differentiate.

The data presented here suggest that cyst cell differentiation is an induced state, and that CySC daughter cells need multiple inputs in order to commit to differentiation (Fig 7). We propose that upon losing access to niche-derived JAK/STAT pathway ligands, CySCs can be primed to differentiate by high levels of PI3K/Tor activity, but do not commit to differentiation until they associate with a gonialblast. Primed CySCs may return to the niche and continue to self-renew if they do not receive this secondary signal. This mechanism to limit differentiation of cyst cells ensures the temporal coordination of cyst cell and gonialblast differentiation.

While much emphasis has been placed on the role of the niche in maintaining stem cell self-renewal, recent work indicates that in the ovary, a separate niche promotes germ cell progression through differentiation [7,48]. Similarly, in the vertebrate intestine, BMP inhibition results in ectopic stem-like cells, indicating a role for BMP signalling in promoting differentiation [8,9]. In both these instances, however, loss of the differentiation signal results in increased self-renewal signalling. Indeed, in the fly ovary, reducing the genetic dose of the GSC self-renewal ligand *dpp* could suppress the accumulation of undifferentiated germ cells caused by disrupting the differentiation niche [48]. By contrast, we show here that Tor is required independently of JAK/STAT signalling to promote cyst cell differentiation, confirming previous work which had shown no upregulation of STAT activity in ectopic CySC-like cells observed upon PI3K/Tor loss-of-function [10]. CySCs deprived of both self-renewal and differentiation-promoting signals maintain a stable stem-like fate, implying that differentiation is not an inevitable consequence of losing access to niche-derived signals. This situation is reminiscent of that described for embryonic stem cells (ESCs), in which self-renewal signals are not necessary for stem cell maintenance if differentiation signals are inhibited [49]. Thus, the ground state of ESCs is to self-renew in the absence of extrinsic signals, and we propose that CySCs similarly have a ground state favourable to self-renewal. This finding suggests that fate decisions may be regulated similarly in embryonic and adult stem cells.

Our results indicate that PI3K/Tor is not simply sufficient for differentiation, however, raising the question as to its role. One attractive model is that PI3K/Tor activity “primes” CySC daughter cells to differentiate, such that Zfh1-positive cells with high PI3K/Tor are able to exit the stem cell pool, but require other signals to do so. We show that cells experiencing high PI3K/Tor activity can go on to maintain CySC fate and self-renew, consistent with the observation that Zfh1-expressing cells in the second row from the hub can give rise to CySCs over time [37]. These cells, although more likely to differentiate, are not necessarily committed to differentiation, and can return to positions adjacent to the hub where PI3K/Tor signalling is lower. This model implies that CySCs exist in multiple states, where some are more biased towards self-renewal, and others towards differentiation, but that cells can transit reversibly between these states [12,50]. As such, the effective stem cell pool consists of both cells directly contacting the hub and those behind them, and the role of PI3K/Tor signalling is to promote the transition to a state biased towards differentiation. How it does so, and what its relevant targets are, is still unclear, although recent work indicates that suppression of autophagy by Tor is important for cyst cell differentiation [39]. Intriguingly, PI3K activity inhibits clonal expansion in mammalian epidermis by promoting progenitor differentiation, but promotes ectopic proliferation and hyperplasia when activated in a widespread manner, suggesting that similar mechanisms may be at play in control of epidermal progenitor differentiation [51,52].

Finally, we present evidence that exit from the cyst stem cell pool depends on the presence of the germline and that PI3K/Tor activity does not regulate this step in differentiation. Previous work had shown that the germline is required to promote cell cycle exit and fate progression in the cyst lineage [46], and conversely, that the encystment of each gonialblast by two cyst cells is required for germ cell differentiation [18,19]. How this coordination and ratio are achieved is still poorly understood; recent work showed that the two stem cells do not synchronise their divisions to produce daughters to co-differentiate [24]. Rather, the abscission, and subsequent differentiation, of the gonialblast from its sibling GSC depends on encystment by cyst cells. The data presented here show that gonialblasts are required for cyst cell differentiation even when Tor is elevated, suggesting that in normal conditions cyst cell differentiation is delayed until a cyst composed of two cyst cells and one gonialblast is assembled. This is consistent with the observation that differentiated cyst cells are always found associated with germ cell cysts. Thus, regulated differentiation of both lineages is essential in order to achieve the coordinated assembly of a cyst. We speculate that PI3K/Tor may prime CySC daughters to interact with neighbouring gonialblasts and promote assembly of this 3-cell cyst. This would explain why relatively high PI3K/Tor activity levels induce CySCs to differentiate, by promoting cyst cell-gonialblast interactions. In the absence of a neighbouring gonialblast, a cell with high PI3K/Tor may come under the influence of niche signals and continue to self-renew. What the final signal promoting the irreversible differentiation of the cyst and exit of the CySC daughters from the stem cell pool is currently unknown. Our model is remarkably similar to that proposed in the case of the mammalian testis, where the germline stem cell pool consists of two populations, one of which is biased towards self-renewal and another towards differentiation, but where individual cells can reversibly move between the populations, until they commit to differentiation [53,54]. This model provides an elegant solution to ensuring that the balance of self-renewal and differentiation is maintained when the differentiation cue is external to the lineage, in the case of the testis, induced by periodic signals of the seminiferous cycle [55]. Our data suggest that, in addition, segregating the signals controlling licensing and commitment to differentiation may ensure the robust coordination of multiple lineages for proper tissue function.

## Materials and methods

### Fly stocks and husbandry

We used the following fly stocks: Oregon-R; *Stat92E^F^; Stat92E^85C9^; FRT^82B^, Tsc1^Q87X^* (gift of N. Tapon)*; FRT^82B^, Tsc1^29^; gig^192^, FRT^80B^; Pten^dj189^, FRT^40A^; UAS-Tsc1 RNAi* (BDSC#54034); *UAS-Dp110* (gift of L. Johnston); *UAS-bam*::*GFP* (gift of R. Lehmann); *UAS-Ci^R^* (*UAS-Ci^76^*, gift of V. Fernandes); *UAS-Ci^act^* (*UAS-Ci5Ncm5*, *UAS-Ci5m30*; gift of D. Kalderon); *tj-Gal4* (Kyoto DGRC#104055); *nos-Gal4-VP16* (gift of R. Lehmann); *C587-Gal4* (gift of R. Lehmann); *spict-Gal4* (Kyoto DGRC#112900); *esg-GFP* (gift of L. Jones); *en-Gal4* (gift of L. Johnston); *tub>stop>Gal80* (BDSC#39213).

Rapamycin feeding was achieved by keeping flies on fly food to which 100 μl of a 4 mM rapamycin stock solution in ethanol was added and air dried. Flies were transferred to fresh vials every 2 days.

Flies were raised at 25°C, except *tj-Gal4* and *C587-Gal4* crosses which were raised at 18°C and adult flies of the correct genotype were maintained at 29°C for 10 days to achieve maximum Gal4 activity.

Positively-marked clones were induced using the MARCM technique [56] with a single 1h-long heat shock at 37°C. Gal80 clones were generated using a flipout cassette in which GAL80 is expressed upon recombination-mediated excision of a stop sequence [57]. Clones were induced using hs-FLP^12^ (BDSC#1929) and with a 30 minute-heat shock at 37°C to maintain low induction rates.

For a full list of genotypes by figure, see S1 Table.

### Immunohistochemistry

Following dissection, testes were fixed in 4% paraformaldehyde in PBS for 15 minutes, then permeabilised twice for 30 minutes in PBS with 0.5% Triton X-100. Samples were then blocked in PBS, 0.2% Triton X-100, 1% Bovine Serum Albumin (PBTB), before being incubated overnight with primary antibodies at 4°C, washed twice for 30 minutes in PBTB, and incubated at room temperature for 2 hours with secondary antibodies. After two washes in PBS, 0.2% Triton X-100 for 30 minutes, testes were mounted on slides with Vectashield medium (Vector labs), and imaged using a Zeiss LSM800, LSM880, or a Leica Sp8 confocal microscope. To detect phosphorylated epitopes, we dissected and fixed in buffer containing phosphatase inhibitors as previously described [26]. EdU incorporation was carried out by incubating dissected samples in Schneider’s medium with 10μM EdU (Invitrogen) for 30 minutes prior to fixing. After primary and secondary antibody steps, samples were incubated in buffer containing 0.1 mM THPTA, 2 mM sodium ascorbate, 1 mM CuSO_4_ and 2.5 μM fluorescently-conjugated picolyl azide (Click Chemistry Tools) for 30 minutes, then briefly washed and mounted for imaging.

We used the following primary antibodies: rabbit anti-Zfh1 (1:5000, gift of R. Lehmann), guinea pig anti-Zfh1 (1:500, gift of J. Skeath), rabbit anti-phospho-4E-BP1 (1:200, Cell Signaling #2855), rabbit anti-phospho-Akt (1:200, Cell Signaling #9271), rabbit anti-phospho-S6k (1:200, Cell Signaling #9209), guinea pig anti-Tj (1:3000, gift of D. Godt), rabbit anti-GFP (1:500, Invitrogen #A-6455), chicken anti-GFP (1:500, Aves Labs #GFP-1010), rat anti-Chinmo (1:50, gift of N. Sokol), rabbit anti-Stat92E (1:1000, gift of E. Bach), rabbit anti-cleaved Dcp-1 (1:500, Cell Signaling #9578); rat anti-Vasa developed by D. Williams and A.C. Spradling (1:20), mouse anti-Fas3 (7G10) developed by C. Goodman (1:20), mouse anti-Eya (10H6), developed by S. Benzer and N.M. Bonini (1:20), rat anti-Ncad (DN-Ex#8), developed by T. Uemura (1:20), mouse anti-Dlg (4F3) developed by C. Goodman (1:20), mouse anti-Arm (N2 7A1 Armadillo), developed by E. Wieschaus were all obtained from the Developmental Studies Hybridoma Bank, created by the NICHD of the NIH and maintained at The University of Iowa, Department of Biology, Iowa City, IA 52242.

### Statistical analysis

Unless otherwise indicated, numbers shown are mean ± standard error. Significance for stem cell number counts was determined using Mann-Whitney tests for pairwise comparison, or non-parametric ANOVA (Kruskal-Wallis test) followed by Dunn’s multiple comparisons test to assess differences between multiple samples. For clone recovery rates, Fisher’s exact test was carried out. All P values were determined using GraphPad Prism software. Plots show individual data points and the lines indicate the mean and 95% confidence interval.

## Supporting information

S1 Fig Hedgehog signalling does not affect Tor activity in CySCs. A-C. Testes from a control animal (A), or expressing the repressor form of the Hh pathway effector Cubitus interruptus (Ci^R^, B) or the activator form (Ci^Act^, C) in somatic cells of the testis, labelled with Zfh1 (magenta, single channel in A’-C’), p4E-BP (cyan, single channel in A”-C”), Eya (yellow, single channel in A”’-C”’) and NCad (white). Although manipulating Hh activity affects the number of Zfh1-expressing cells, the pattern of p4E-BP was not altered, as CySCs adjacent to the hub had low levels of p4E-BP (white arrowheads), while cells further distal from the hub displayed high levels of p4E-BP (yellow arrowheads). The hub is indicated with NCad expression or a dotted line. Scale bar in all panels represents 20 μm.
(TIF)

S2 Fig *Tsc1* mutant clones display normal levels of adhesion and do not die. A,B. Control (A) or *Tsc1* mutant (B) clones at 2 dpci marked by GFP expression (yellow, single channel A’,B’) and labelled with antibodies against Armadillo (Arm, cyan, single channel A”,B”) and Tj (magenta, single channel A”’, B”’). Both control clones and mutants contact the hub with membrane extensions enriched in Arm (arrows). Additionally, both control and mutant clones display a characteristic morphology when differentiating and enclosing germ cells (red arrowheads), and show enrichment of Arm along the interface with the enclosed germ cell. C,D. Control (C) or *Tsc1* mutant (D) clones at 2 dpci marked by GFP expression (yellow) and labelled with antibodies against the apoptosis marker Dcp-1 (cyan, single channel C’,D’), Tj (magenta) and Fas3 (white). Dcp-1-positive cells are visible outside the clones (arrowheads). The hub is indicated with Fas3 or Arm expression or a dotted line. Scale bar in all panels represents 20 μm.
(TIF)

S3 Fig p4E-BP expression colocalises with *spict-Gal4*. GFP (cyan) was driven by *spict-Gal4* for 20 h by raising flies with a *tub>Gal80*^*ts*^ transgene at 18°C and incubating them at 29°C overnight to induce expression. GFP is detected in cells two rows away from the hub (arrows), colocalising with high p4E-BP expression (yellow). Tj (magenta) labels somatic cell nuclei, Ncad (white) labels the hub. Scale bar represents 20μm.
(TIF)

S4 Fig Dp110 over-expression and Tsc1 knockdown in wing discs show expected pathway activity. Third instar larval wing imaginal discs expressing GFP (yellow) in the posterior compartment under the control of *engrailed (en)-Gal4*. Compared to control discs (A,C), expression of Dp110 (C) or Tsc1 RNAi (D) results in increased growth of the posterior compartment, seen by a decreased density of cell nuclei (cyan). Dp110 expression leads to increased staining for phosphorylated Akt (pAkt, magenta in A,B), while Tsc1 knockdown results in higher phosphorylated Ribosomal protein S6 kinase (pS6k, magenta in C,D), canonical targets of Insulin and Tor signalling, respectively. Scale bar in all panels represents 50μm.
(TIF)

S5 Fig CySCs are still present when Tor is hyperactivated. Control (A,B), Dp110 over-expression (C,D) or Tsc1 knockdown (E,F) in somatic cells of the testis. A,C,E. Testes labelled with Zfh1 (green), Eya and Fas3 (cyan) and EdU (magenta, single channel in A’,C’,E’), showing EdU incorporation in Zfh1-positive cells adjacent to the hub (arrowheads). B,D,F. Testes labelled with antibodies against Stat92E (yellow, single channel in B’,D’,F’), Chinmo (cyan, single channel in B”,D”,F”), Tj (magenta) and Eya and Fas3 (white). Stat92E is detected only in CySCs around the hub (arrowheads). Chinmo is detected in the hub, CySCs and early differentiating cyst cells, as well as early germ cells and spermatocytes. Chinmo is downregulated in Eya-positive differentiated cyst cells (arrows). The hub is indicated with Fas3 expression or a dotted line. Scale bar in all panels represents 20 μm.
(TIF)

S6 Fig Germline ablation results in ectopic CySC-like cells but not JAK/STAT signalling. Control (A,B) or germline-ablated testes (C,D) labelled with antibodies against Tj (magenta) and Fas3 (cyan). A,C. Chinmo (yellow, single channel A’,C’) expression is downregulated in differentiating cyst cells in control (B, arrows), but its expression is maintained far from the hub when the germline is absent (C, arrows). B,D. Stat92E is detected in GSCs and CySCs adjacent to the hub, and occasionally in gonialblasts in controls (B). In germline-ablated testes (C), Stat92E is detected in Tj-positive cells up to three cell diameters from the hub, but not in cells more distant from the hub, suggesting that ectopic JAK/STAT signalling cannot account for continued self-renewal away from the hub in this condition. The hub is indicated with Fas3 expression or a dotted line. Scale bar in all panels represents 20 μm.
(TIF)

S1 Table List of experimental genotypes. (DOCX)
