# Supplementary figures and images for: Germ cells commit somatic stem cells to differentiation following priming by PI3K/Tor activity in the Drosophila testis
Source: PLoS Genet. 2021 Dec 13;17(12):e1009609. doi: 10.1371/journal.pgen.1009609 (PMC8699969; doi:10.1371/journal.pgen.1009609)

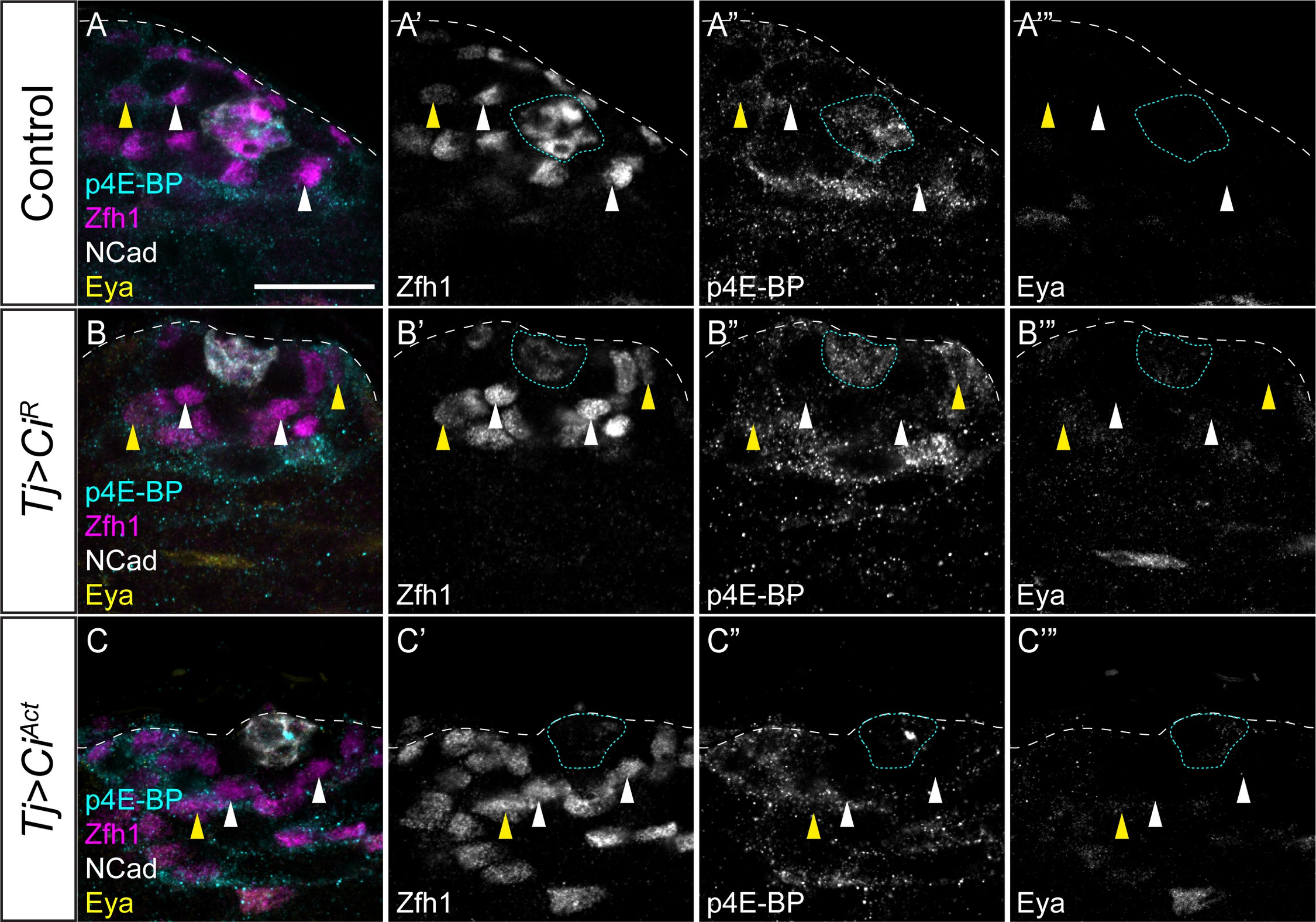

Supplement: S1 Fig — A-C. Testes from a control animal (A), or expressing the repressor form of the Hh pathway effector Cubitus interruptus (CiR, B) or the activator form (CiAct, C) in somatic cells of the testis, labelled with Zfh1 (magenta, single channel in A’-C’), p4E-BP (cyan, single channel in A”-C”), Eya (yellow, single channel in A”’-C”’) and NCad (white). Although manipulating Hh activity affects the number of Zfh1-expressing cells, the pattern of p4E-BP was not altered, as CySCs adjacent to the hub had low levels of p4E-BP (white arrowheads), while cells further distal from the hub displayed high levels of p4E-BP (yellow arrowheads). The hub is indicated with NCad expression or a dotted line. Scale bar in all panels represents 20 μm. (TIF) [file pgen.1009609.s001.tif]

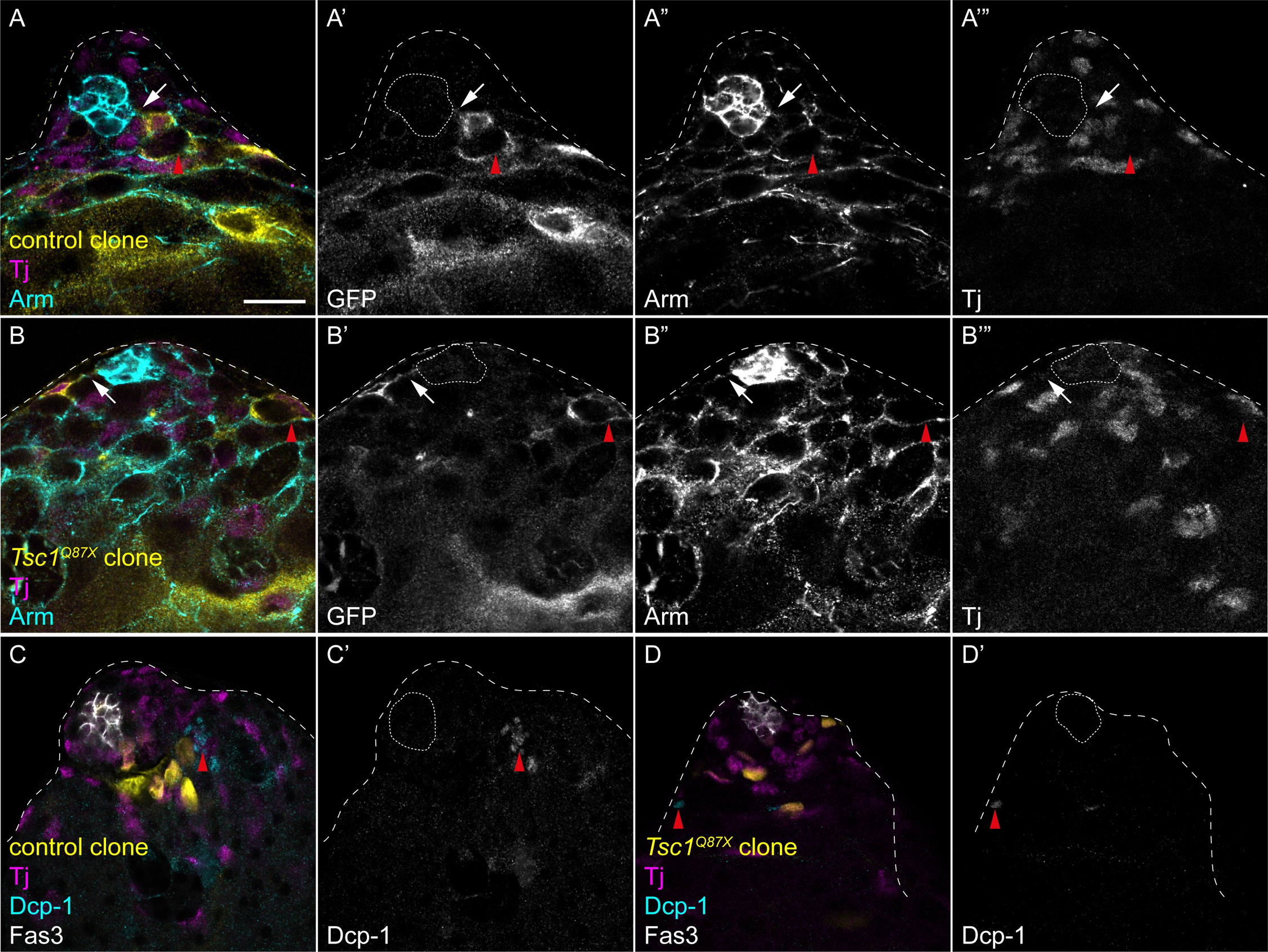

Supplement: S2 Fig — A,B. Control (A) or Tsc1 mutant (B) clones at 2 dpci marked by GFP expression (yellow, single channel A’,B’) and labelled with antibodies against Armadillo (Arm, cyan, single channel A”,B”) and Tj (magenta, single channel A”’, B”’). Both control clones and mutants contact the hub with membrane extensions enriched in Arm (arrows). Additionally, both control and mutant clones display a characteristic morphology when differentiating and enclosing germ cells (red arrowheads), and show enrichment of Arm along the interface with the enclosed germ cell. C,D. Control (C) or Tsc1 mutant (D) clones at 2 dpci marked by GFP expression (yellow) and labelled with antibodies against the apoptosis marker Dcp-1 (cyan, single channel C’,D’), Tj (magenta) and Fas3 (white). Dcp-1-positive cells are visible outside the clones (arrowheads). The hub is indicated with Fas3 or Arm expression or a dotted line. Scale bar in all panels represents 20 μm. (TIF) [file pgen.1009609.s002.tif]

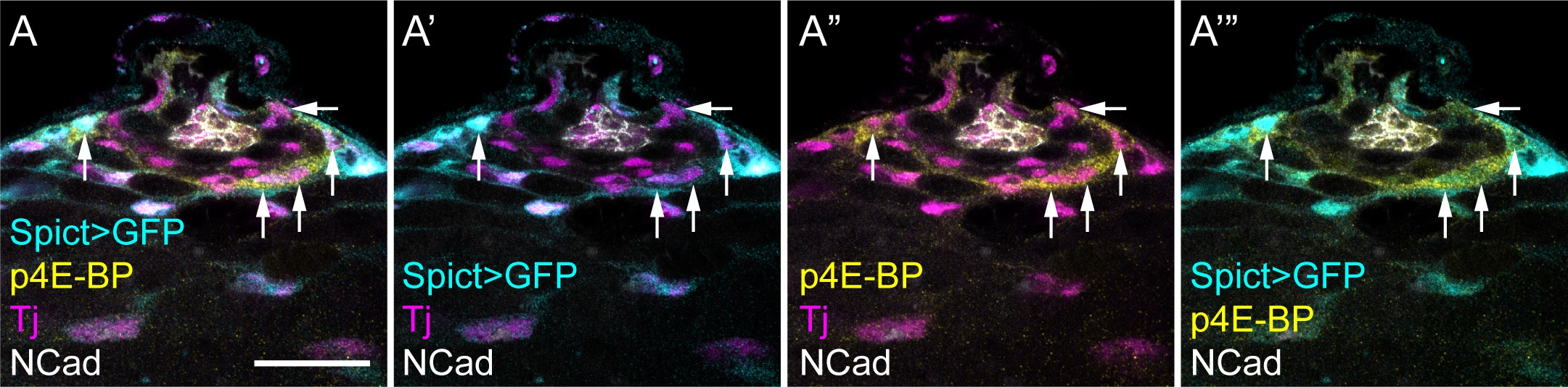

Supplement: S3 Fig — GFP (cyan) was driven by spict-Gal4 for 20 h by raising flies with a tub>Gal80ts transgene at 18°C and incubating them at 29°C overnight to induce expression. GFP is detected in cells two rows away from the hub (arrows), colocalising with high p4E-BP expression (yellow). Tj (magenta) labels somatic cell nuclei, Ncad (white) labels the hub. Scale bar represents 20μm. (TIF) [file pgen.1009609.s003.tif]

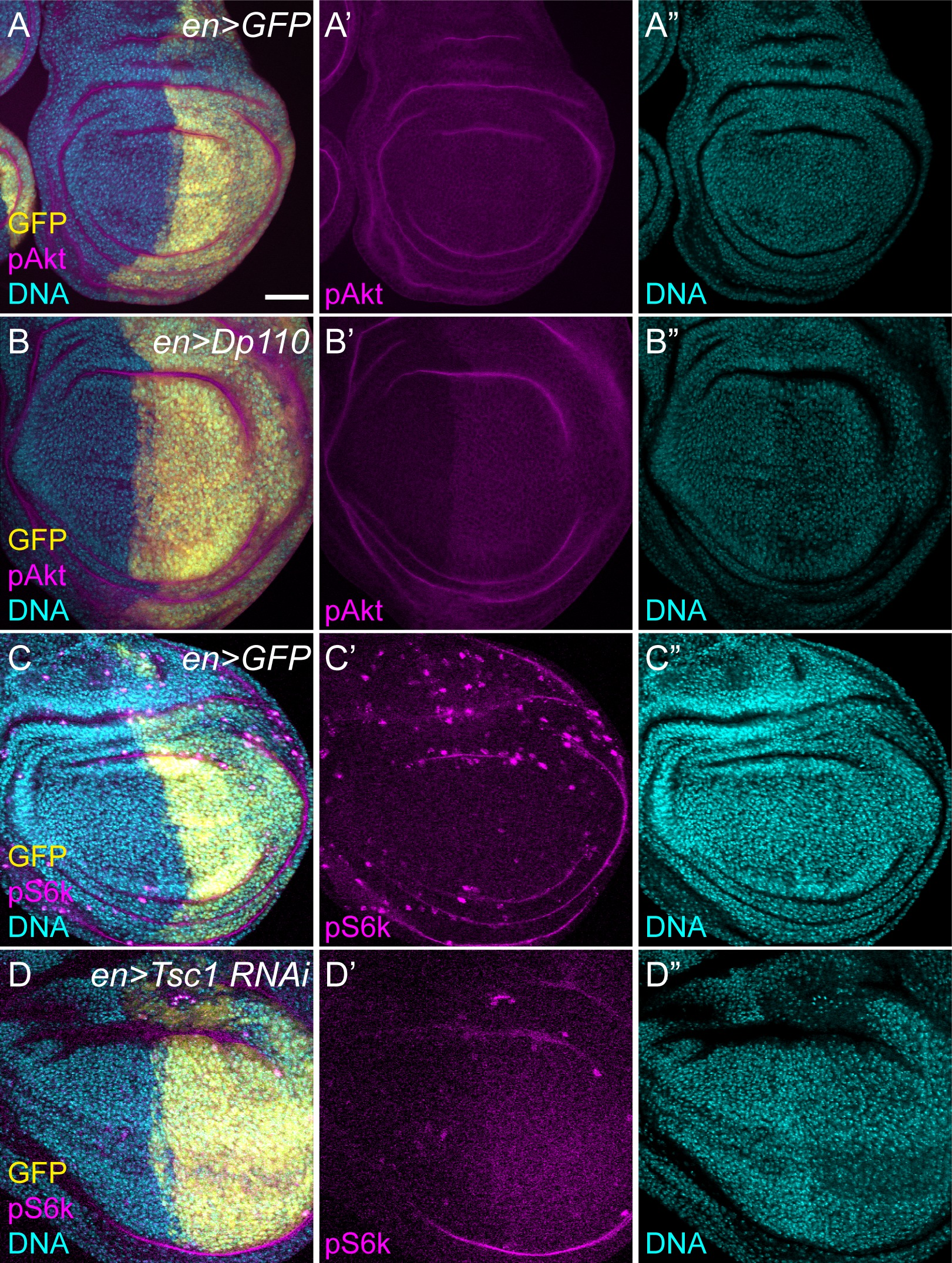

Supplement: S4 Fig — Third instar larval wing imaginal discs expressing GFP (yellow) in the posterior compartment under the control of engrailed (en)-Gal4. Compared to control discs (A,C), expression of Dp110 (C) or Tsc1 RNAi (D) results in increased growth of the posterior compartment, seen by a decreased density of cell nuclei (cyan). Dp110 expression leads to increased staining for phosphorylated Akt (pAkt, magenta in A,B), while Tsc1 knockdown results in higher phosphorylated Ribosomal protein S6 kinase (pS6k, magenta in C,D), canonical targets of Insulin and Tor signalling, respectively. Scale bar in all panels represents 50μm. (TIF) [file pgen.1009609.s004.tif]

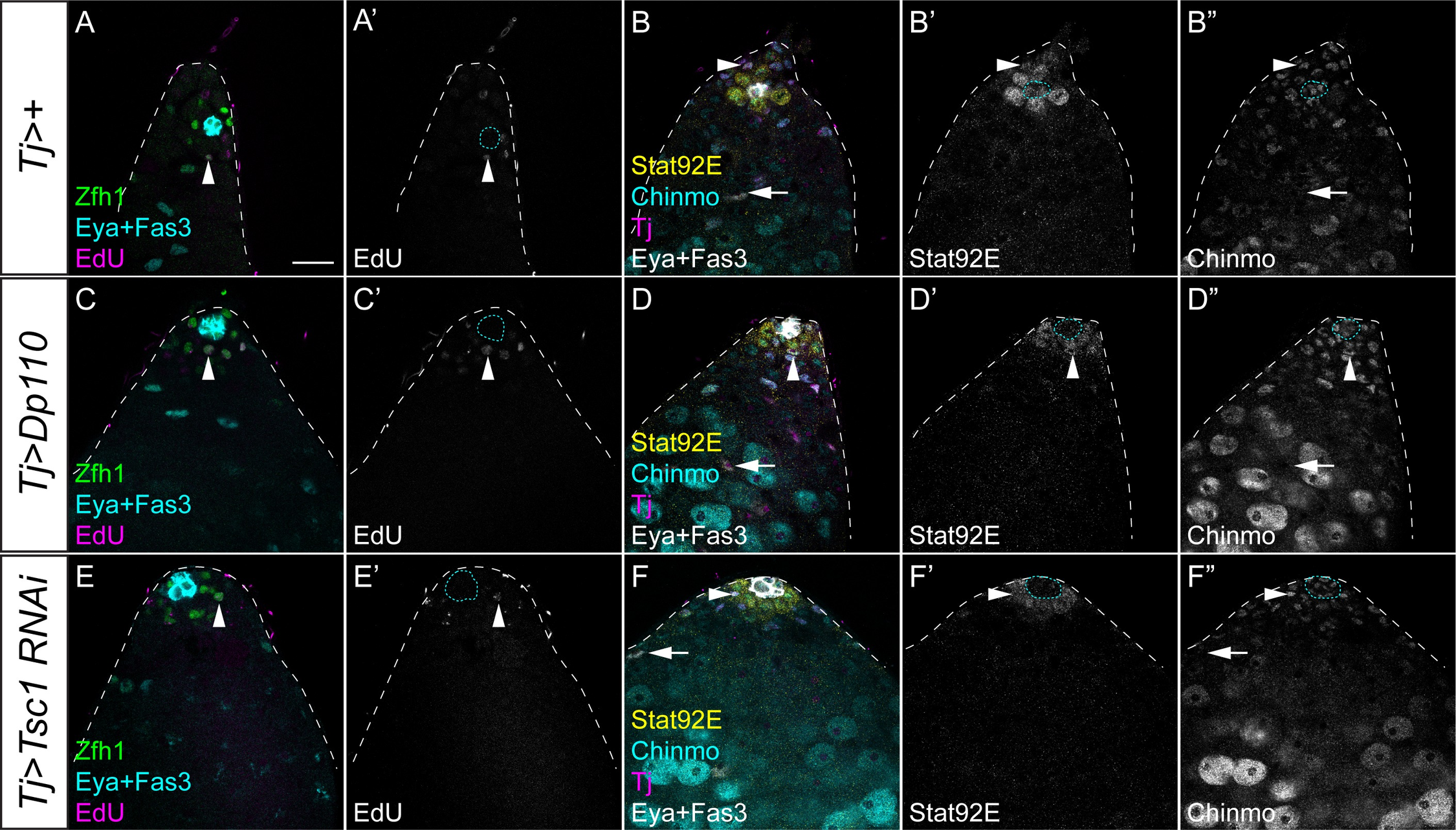

Supplement: S5 Fig — Control (A,B), Dp110 over-expression (C,D) or Tsc1 knockdown (E,F) in somatic cells of the testis. A,C,E. Testes labelled with Zfh1 (green), Eya and Fas3 (cyan) and EdU (magenta, single channel in A’,C’,E’), showing EdU incorporation in Zfh1-positive cells adjacent to the hub (arrowheads). B,D,F. Testes labelled with antibodies against Stat92E (yellow, single channel in B’,D’,F’), Chinmo (cyan, single channel in B”,D”,F”), Tj (magenta) and Eya and Fas3 (white). Stat92E is detected only in CySCs around the hub (arrowheads). Chinmo is detected in the hub, CySCs and early differentiating cyst cells, as well as early germ cells and spermatocytes. Chinmo is downregulated in Eya-positive differentiated cyst cells (arrows). The hub is indicated with Fas3 expression or a dotted line. Scale bar in all panels represents 20 μm. (TIF) [file pgen.1009609.s005.tif]

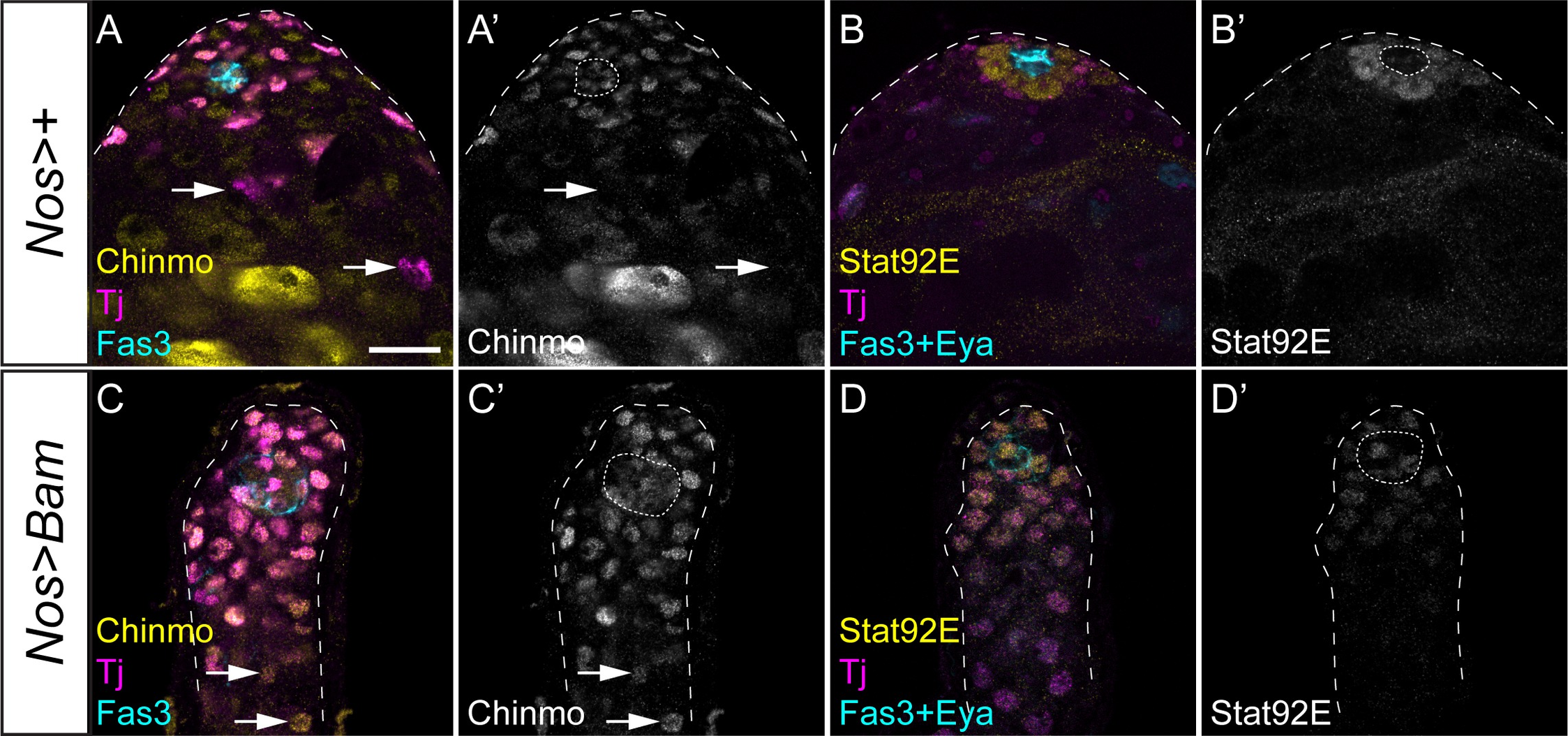

Supplement: S6 Fig — Control (A,B) or germline-ablated testes (C,D) labelled with antibodies against Tj (magenta) and Fas3 (cyan). A,C. Chinmo (yellow, single channel A’,C’) expression is downregulated in differentiating cyst cells in control (B, arrows), but its expression is maintained far from the hub when the germline is absent (C, arrows). B,D. Stat92E is detected in GSCs and CySCs adjacent to the hub, and occasionally in gonialblasts in controls (B). In germline-ablated testes (C), Stat92E is detected in Tj-positive cells up to three cell diameters from the hub, but not in cells more distant from the hub, suggesting that ectopic JAK/STAT signalling cannot account for continued self-renewal away from the hub in this condition. The hub is indicated with Fas3 expression or a dotted line. Scale bar in all panels represents 20 μm. (TIF) [file pgen.1009609.s006.tif]
