# Supplementary material for: Germ cells commit somatic stem cells to differentiation following priming by PI3K/Tor activity in the Drosophila testis
Source: PLoS Genet. 2021 Dec 13;17(12):e1009609. doi: 10.1371/journal.pgen.1009609 (PMC8699969; doi:10.1371/journal.pgen.1009609)
Supplement: S1 Table — (DOCX) [file pgen.1009609.s007.docx]

**S1 Table: List of experimental genotypes.**

| **Figure panel** | **Genotype** |
| --- | --- |
| 1C | *w/Y;; FRT^82B^, Stat92E^85c9^/+* |
| 1D | *w/Y;; FRT^82B^, Stat92E^85c9^/FRT^82B^, Stat92E^F^* |
| 1E | *w/Y;; FRT^82B^, Stat92E^85c9^/+* |
| 1F | *w/Y;; FRT^82B^, Stat92E^85c9^/FRT^82B^, Stat92E^F^* |
| 1G | *w/Y;; FRT^82B^, Stat92E^85c9^/+* |
| 1H | *w/Y;; FRT^82B^, Stat92E^85c9^/FRT^82B^, Stat92E^F^* |
| 1I | *w/Y;; FRT^82B^, Stat92E^85c9^/FRT^82B^, Stat92E^F^* |
| 2A | *y,w,hsflp^122^, UAS-nlsGFP/Y;; Tub>Gal4, FRT^82B^ Tub>Gal80/FRT^82B^* |
| 2B | *y,w,hsflp^122^, UAS-nlsGFP/Y;; Tub>Gal4, FRT^82B^ Tub>Gal80/FRT^82B^* |
| 2C | *y,w,hsflp^122^, UAS-nlsGFP/Y;; Tub>Gal4, FRT^82B^ Tub>Gal80/FRT^82B^ Tsc1^Q87X^* |
| 2D | *y,w,hsflp^122^, UAS-nlsGFP/Y;; Tub>Gal4, FRT^82B^ Tub>Gal80/FRT^82B^ Tsc1^Q87X^* |
| 2E | *y,w,hsflp^122^, UAS-nlsGFP/Y;; Tub>Gal80 FRT^80B^, Tub>Gal4/FRT^80B^* |
| 2F | *y,w,hsflp^122^, UAS-nlsGFP/Y;; Tub>Gal80 FRT^80B^, Tub>Gal4/FRT^80B^* |
| 2G | *y,w,hsflp^122^, UAS-nlsGFP/Y;; Tub>Gal80 FRT^80B^, Tub>Gal4/gig^192^ FRT^80B^* |
| 2H | *y,w,hsflp^122^, UAS-nlsGFP/Y;; Tub>Gal80 FRT^80B^, Tub>Gal4gig^192^/FRT^80B^* |
| 2I | *y,w,hsflp^122^, UAS-nlsGFP/Y;; Tub>Gal4, FRT^82B^ Tub>Gal80/FRT^82B^* |
| 2J | *y,w,hsflp^122^, UAS-nlsGFP/Y;; Tub>Gal4, FRT^82B^ Tub>Gal80/FRT^82B^ Tsc1^Q87X^* |
| 2K | *y,w,hsflp^122^, UAS-nlsGFP/Y;; Tub>Gal80 FRT^80B^, Tub>Gal4/FRT^80B^* |
| 2L | *y,w,hsflp^122^, UAS-nlsGFP/Y;; Tub>Gal80 FRT^80B^, Tub>Gal4/gig^192^ FRT^80B^* |
| 2M | As above, 2A-2H |
| 3A | *yw/Y; Tj-Gal4/+;* |
| 3B | *C587-Gal4/Y; esg-GFP/+* |
| 3C | *yw/Y;Tj-Gal4/+; UAS-Dp110/+* |
| 3D | *C587-Gal4; esg-GFP/+; UAS-Dp110/+* |
| 3E | *yw/Y; Tj-Gal4/UAS-Tsc1 RNAi;* |
| 3F | *C587-Gal4/Y; esg-GFP/UAS-Tsc1 RNAi;* |
| 3G | *yw/Y; Tj-Gal4/+;* |
| 3H | *yw/Y;Tj-Gal4/+; UAS-Dp110/+* |
| 3I | *yw/Y; Tj-Gal4/UAS-Tsc1 RNAi;* |
| 4A | *C587-Gal4/Y; ubi-GFP FRT^40A^/FRT^40A^;* |
| 4B | *C587-Gal4/Y; ubi-GFP FRT^40A^/FRT^40A^;* |
| 4C | *C587-Gal4/Y; ubi-GFP FRT^40A^/pten^dj189^ FRT^40A^;* |
| 4D | *C587-Gal4/Y; ubi-GFP FRT^40A^/pten^dj189^ FRT^40A^;* |
| 4E | *C587-Gal4/Y; ubi-GFP FRT^40A^/FRT^40A^; UAS-Dp110/+* |
| 4F | *C587-Gal4/Y; ubi-GFP FRT^40A^/FRT^40A^; UAS-Dp110/+* |
| 4G | *C587-Gal4/Y; ubi-GFP FRT^40A^/pten^dj189^ FRT^40A^; UAS-Dp110/+* |
| 4H | *C587-Gal4/Y; ubi-GFP FRT^40A^/pten^dj189^ FRT^40A^; UAS-Dp110/+* |
| 4I | As above, 4A-4H |
| 5B | *y,w,hsflp^12^/Y; Tj-Gal4, UAS-GFP/+; Tub>Stop>Gal80/+* |
| 5C | *y,w,hsflp^12^/Y; Tj-Gal4, UAS-GFP/UAS-Dp110; Tub>Stop>Gal80/+* |
| 5D | As above, 5B-5C |
| 6A | *y,w,hsflp^122^/Y; Nos-Gal4::Vp16/+;* |
| 6B | *y,w,hsflp^122^/Y; Nos-Gal4::VP16/UAS-bam::GFP;* |
| 6C | *y,w,hsflp^122^/Y; Nos-Gal4::VP16/+; FRT^82B^ ubi-GFP/FRT^82B^* |
| 6D | *y,w,hsflp^122^/Y; Nos-Gal4::VP16/+; FRT^82B^ ubi-GFP/FRT^82B^ Tsc1^29^* |
| 6E | *y,w,hsflp^122^/Y; Nos-Gal4::VP16/UAS-bam::GFP; FRT^82B^ ubi-GFP/FRT^82B^* |
| 6F | *y,w,hsflp^122^/Y; Nos-Gal4/UAS-bam::GFP; FRT^82B^ ubi-GFP/FRT^82B^ Tsc1^29^* |
| 6G | As above, 6C-6F |
|  |  |
| S1A | *yw/Y; Tj-Gal4/+;* |
| S1B | *yw/Y; Tj-Gal4/+; UAS-Ci^76^/+* |
| S1C | *yw/Y; Tj-Gal4/+; UAS-Ci5Ncm5, UAS-Ci5m30* |
| S2A | *y,w,hsflp^122^, UAS-CD8::GFP/Y;; Tub>Gal4, FRT^82B^ Tub>Gal80/FRT^82B^* |
| S2B | *y,w,hsflp^122^, UAS-CD8::GFP/Y;; Tub>Gal4, FRT^82B^ Tub>Gal80/FRT^82B^ Tsc1^Q87X^* |
| S2C | *y,w,hsflp^122^, UAS-nlsGFP/Y;; Tub>Gal4, FRT^82B^ Tub>Gal80/FRT^82B^* |
| S2D | *y,w,hsflp^122^, UAS-nlsGFP/Y;; Tub>Gal4, FRT^82B^ Tub>Gal80/FRT^82B^ Tsc1^Q87X^* |
| S3A | *w; Spict^NP2367^-Gal4/+; Tub>Gal80^ts^/+* |
| S4A | *w; en-Gal4, UAS-GFP/+* |
| S4B | *w; en-Gal4, UAS-GFP/+; UAS-Dp110* |
| S4C | *w; en-Gal4, UAS-GFP/+* |
| S4D | *w; en-Gal4, UAS-GFP/ UAS-Tsc1 RNAi* |
| S5A | *yw/Y; Tj-Gal4/+;* |
| S5B | *yw/Y; Tj-Gal4/+;* |
| S5C | *yw/Y;Tj-Gal4/+; UAS-Dp110/+* |
| S5D | *yw/Y;Tj-Gal4/+; UAS-Dp110/+* |
| S5E | *yw/Y; Tj-Gal4/UAS-Tsc1 RNAi;* |
| S5F | *yw/Y; Tj-Gal4/UAS-Tsc1 RNAi;* |
| S6A | *y,w,hsflp^122^/Y; Nos-Gal4::Vp16/+;* |
| S6B | *y,w,hsflp^122^/Y; Nos-Gal4::Vp16/+;* |
| S6C | *y,w,hsflp^122^/Y; Nos-Gal4::VP16/UAS-bam::GFP;* |
| S6D | *y,w,hsflp^122^/Y; Nos-Gal4::VP16/UAS-bam::GFP;* |
